# Long-term maintenance of patient-specific characteristics in tumoroids from six cancer indications

**DOI:** 10.1038/s41598-025-86979-9

**Published:** 2025-01-31

**Authors:** Colin D. Paul, Chris Yankaskas, Pradip Shahi Thakuri, Brittany Balhouse, Shyanne Salen, Amber Bullock, Sylvia Beam, Anthony Chatman, Sybelle Djikeng, Xiaoyu Jenny Yang, Garrett Wong, Isha Dey, Spencer Holmes, Abigail Dockey, Lindsay Bailey-Steinitz, Lina Zheng, Weizhong Li, Vivek Chandra, Jakhan Nguyen, Jason Sharp, Erik Willems, Mark Kennedy, Matthew R. Dallas, David Kuninger

**Affiliations:** 1 Thermo Fisher Scientific, Frederick, MD USA; 2 Thermo Fisher Scientific, Carlsbad, CA USA; 3 Thermo Fisher Scientific, Bengaluru, Karnataka India

**Keywords:** Cancer models, Cell growth

## Abstract

Tumoroids, sometimes referred to as cancer organoids, are patient-derived cancer cells grown as 3D, self-organized multicellular structures that maintain key characteristics (e.g., genotype, gene expression levels) of the tumor from which they originated. These models have emerged as valuable tools for studying tumor biology, cytotoxicity, and response of patient-derived cells to cancer therapies. However, the establishment and maintenance of tumoroids has historically been challenging, labor intensive, and highly variable from lab to lab, hindering their widespread use. Here, we characterize the establishment and/or expansion of colorectal, lung, head and neck, breast, pancreas, and endometrial tumoroids using the standardized, serum-free Gibco OncoPro Tumoroid Culture Medium. Newly derived tumoroid lines (*n* = 20) were analyzed by targeted genomic profiling and RNA sequencing and were representative of tumor tissue samples. Tumoroid lines were stable for over 250 days in culture and freeze-thaw competent. Previously established tumoroid lines were also transitioned to OncoPro medium and exhibited, on average, similar growth rates and conserved donor-specific characteristics when compared to original media systems. Additionally, OncoPro medium was compatible with both embedded culture in extracellular matrix and growth in a suspension format for facile culture and scale up. An example application of these models for assessing the cytotoxicity of a natural killer cell line and primary natural killer cells over time and at various doses demonstrated the compatibility of these models with assays used in compound and cell therapy development. We anticipate that the standardization and versatility of this approach will have important benefits for basic cancer research, drug discovery, and personalized medicine and help make tumoroid models more accessible to the cancer research community.

**Supplementary Information:**

The online version contains supplementary material available at 10.1038/s41598-025-86979-9.

## Introduction

A common challenge facing cancer researchers is the need for in vitro cancer models representative of the mutational patterns and gene expression levels observed in patient populations. Indeed, differences in mutational burden and gene expression between patient tumors and immortalized cancer cell lines^1–5^ may contribute to drug development bottlenecks in which agents effective in vitro do not translate to complex and multifactorial clinical settings. As a result, there has been increasing interest in utilizing more physiologically relevant cancer models in pre-clinical research, for a more efficient treatment development pipeline^6^ and to reduce animal testing^7^. Tumoroids, sometimes referred to as cancer organoids, are patient-derived cancer cells that self-organize into three-dimensional (3D) structures in culture and preserve key phenotypic and genotypic features of patient cancers. Our own work comparing two-dimensional (2D) colorectal cancer cell lines with randomly selected primary colorectal cancer donor samples and non-matched colorectal tumoroids demonstrated that the 2D lines tested harbored higher numbers of clinically relevant mutations and had higher tumor mutational burdens compared to tumors and tumoroid lines (Supplementary Fig. S1). In addition, quantification of gene expression levels followed by hierarchical clustering revealed that the 2D lines tested clustered separately from the primary tumors, which instead clustered together with tumoroids (Supplementary Fig. S1). These observations emphasize that 2D lines are, in some cases, hypermutated and less representative of patient cancer gene expression patterns compared to tumoroid models. Therefore, tumoroids hold great promise in both pre-clinical and translational research. Specifically, these physiologically-relevant models could reveal aspects of tumor biology that are not recapitulated by immortalized cancer cell lines and could help increase the efficiency of treatment discovery pipelines^8^, and, potentially, identify effective patient treatments through functional precision medicine approaches^9^.

While the value of tumoroid culture in cancer research is apparent, the complexity of the approach has limited its adoption. Many methods for tumoroid culture rely on complicated 3D culture handling and medium production protocols^10^, lack of standardization amongst bespoke media recipes (“homebrew” media systems), reliance on conditioned medium^11–13^, and growth competition with benign cell types^14–16^. In this study, we demonstrate the capability of the serum-free, conditioned medium-free Gibco OncoPro Tumoroid Culture Medium (referred to throughout as “OncoPro medium”) to support derivation and long-term culture of colorectal, lung, breast, and endometrial tumoroid models, wherein the patient-specific characteristics identified from primary tumor tissue are recapitulated throughout the culture period. Specifically, the system is composed of Advanced DMEM/F-12 + 1X GlutaMAX basal media, 1X B-27 supplement, a bovine serum albumin (BSA) supplement, and a proprietary OncoPro Supplement, which are purchased as a kit and combined to form the complete serum-free OncoPro medium (see Materials and Methods and Supplementary Table S1). The system relies on this conserved base OncoPro medium, with the addition of 1–2 niche factors for some indications to support growth. For example, Heat Stable FGF-10 (HS FGF-10) is added to OncoPro medium to support lung tumoroid culture; see below and Supplementary Table S1 for details on additional cancer indications. Tumoroid culture was also performed in the presence of antibiotics and the ROCK inhibitor Y27632. Tumoroid expansion in the system is compatible with 3D encapsulation in basement membrane extract (BME) domes but also amenable to a suspension culture approach where the cells float freely in the presence of diluted BME to provide extracellular matrix (ECM) cues. Suspension tumoroid culture reduces BME consumption and simplifies handling to facilitate scale up and use in high-throughput applications. We show the compatibility of this novel media system and suspension culture workflow with the expansion of colorectal, lung, head and neck, breast, and pancreas tumoroid lines that had been previously cultured in homebrew media formulations, while maintaining the distinctive mutational and gene expression profiles of each cell model. Finally, to illustrate the utility of tumoroid models in preclinical cell therapy research, we developed a tumoroid line that stably expresses green fluorescent protein (GFP) and used these tumoroids to interrogate the killing potential of primary natural killer (NK) cells and an immortalized NK cell line (NK-92). Together, our results illustrate how OncoPro medium is broadly effective in maintaining donor-specific characteristics during in vitro tumoroid cell culture.

## Results

### Derivation of tumoroid lines from primary cancer samples across multiple indications using a conserved base medium formulation

**Fig. 1:**
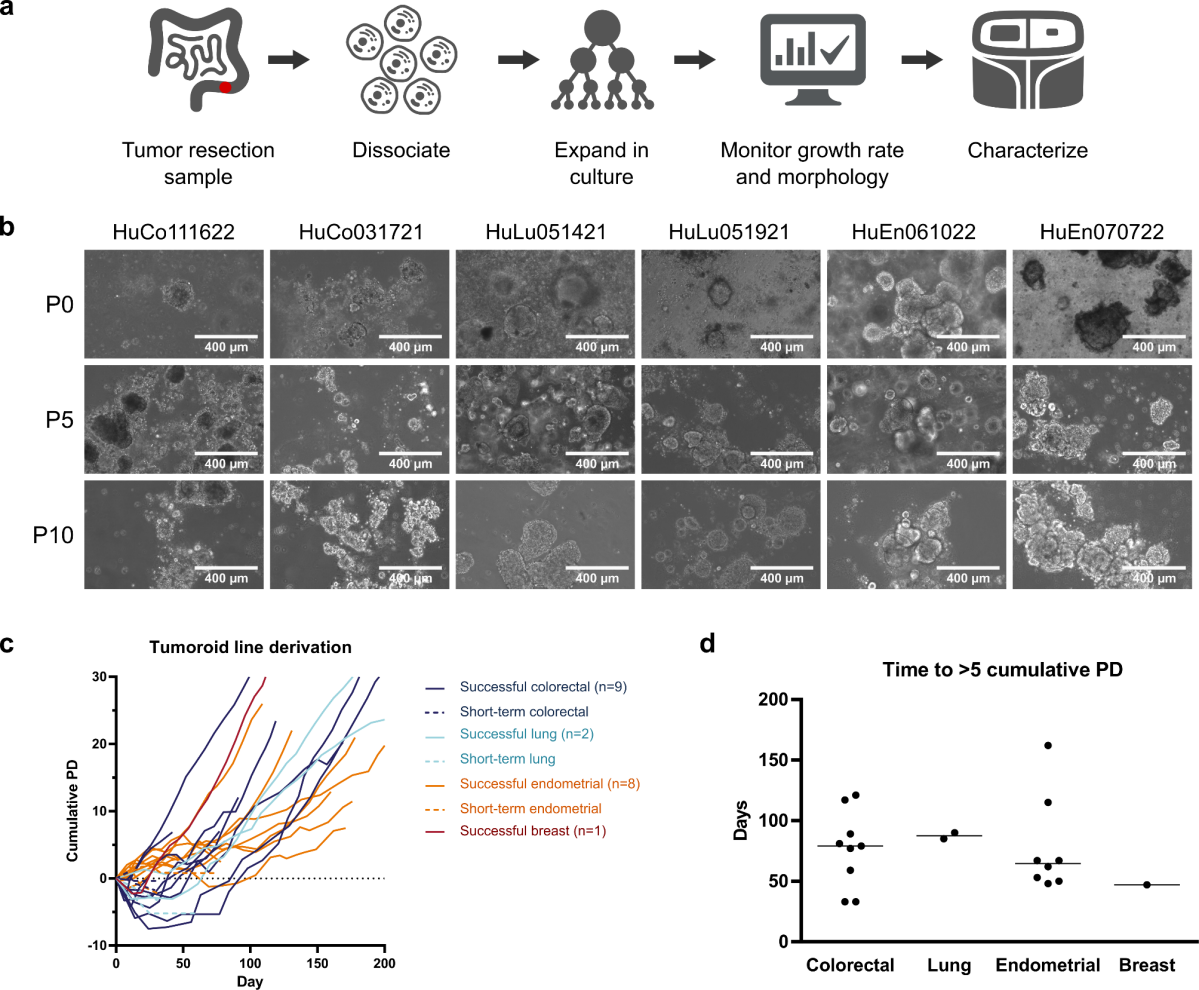
Tumoroid lines can be derived from donor material across several cancer indications. **(a)** Schematic of tumoroid establishment and characterization. Donor tumor material was processed, cultured, and analyzed for fidelity to the original cells sampled at the time of collection by next-generation sequencing. **(b)** Representative images of tumoroid lines at passages (P) 0, 5, and 10. P0 images were acquired after 1–2 weeks of culture. P0 and P5 images represent a mix of embedded and suspension samples. All P10 images were suspension cultures. HuCo = human colorectal cancer; HuLu = human lung cancer; HuEn = human endometrial cancer. Scale bar = 400 μm. **(c)** Cumulative population doublings (PD) over time during attempted derivation of colorectal, lung, endometrial, and breast tumoroid lines. Dashed lines indicate short-term cultures that formed tumoroids initially but failed to expand in vitro over the long term. **(d)** Time (in days) for successfully established tumoroid lines to reach 5 PD based on initial cell seeding number for various indications. Each point represents one donor, and each line is plotted at the median. For panels (c) and (d), successful colorectal donors – HuCo1044, HuCo3209, HuCo021320, HuCo031721, HuCo111622, HuCo080922, HuCo090123, HuCo083123, HuCo100523; successful lung donors – HuLu051421, HuLu051921; successful breast donor – HuBr102622; successful endometrial donors – HuEn033122, HuEn041521, HuEn061022, HuEn070722, HuEn022222, HuEn052622B, HuEn052622A, HuEn062322.

To demonstrate the effectiveness of our approach for tumoroid derivation, we tested whether the OncoPro medium formulation supported generation of novel patient-derived cultures from viable cancer cells. Dissociated cells, either from commercially available cryopreserved material or following enzymatic dissociation of fresh primary tumor surgical resection samples, were cultured in OncoPro medium (Fig. 1a). Samples from the initial material were also collected for subsequent genetic and transcriptomic analysis. No red blood cell lysis or cell selection/sorting was performed prior to placing all recovered cells from dissociated tumor samples into culture, sampling dissociated cell populations for flow cytometry, or collecting samples for sequencing. The indications represented in the derivation experiments were colorectal, lung, breast, and endometrial cancers. For lung cancer samples, the base OncoPro medium formulation was supplemented with HS FGF-10, while medium for breast and endometrial cancer tumoroids was supplemented with HS FGF-10 and beta-estradiol (Supplementary Table S1). Our derivation workflow typically utilized a hybrid approach combining embedded culture in BME hydrogel domes for the first several passages (typically ~ 5), followed by scale up in a suspension culture format wherein 2% (v/v) basement membrane extract was added to OncoPro medium in non-tissue culture treated cell culture dishes and flasks to maintain the self-organized 3D morphologies characteristic of tumoroids.

Greater than 85% of samples initially formed 3D tumoroid structures and exhibited a range of donor-specific morphologies (Fig. 1b), which were retained for the duration of culture in either embedded or suspension formats. Tumoroids were subcultured by dissociating multicellular 3D structures when they reached an average diameter of 100–300 μm and seeding a mixture of single cells and small cell clusters (Supplementary Fig. S2); samples that did not grow as large were passaged after a period of 14 days. Occasionally, overall cell number would decline during the first few passages in culture (Fig. 1c). Approximately half of cultures grew stably in the long term, which we defined as expanding consistently for more than 5 passages and exceeding 5 cumulative population doublings from the initial number of cells seeded (including any non-cancer cells present in the initial sample; Fig. 1c). These stringent cutoffs were good predictors of whether a tumoroid culture would continue to grow consistently and were confirmed in 2 colorectal and 2 lung samples, which grew for over 275 days. Select examples of unsuccessful derivation (tumoroids reforming for only a few passages) are also given (Fig. 1c). Derivation of cultures to these standards typically required 50–120 days (Fig. 1d). Based on these derivation criteria, 9 colorectal tumoroid lines, 2 lung tumoroid lines, 1 breast tumoroid line, and 8 endometrial tumoroid lines were successfully established and cryopreserved for subsequent use. Donor characteristics for derived tumoroid lines and information on whether cultures were initiated from viably cryopreserved dissociated tumor cells or fresh tumor resection samples (which were dissociated in our lab and then cultured) are summarized in Supplementary Table S2.

### Genomic characteristics of original tumor cells are retained in patient-derived tumoroid cultures

**Fig. 2:**
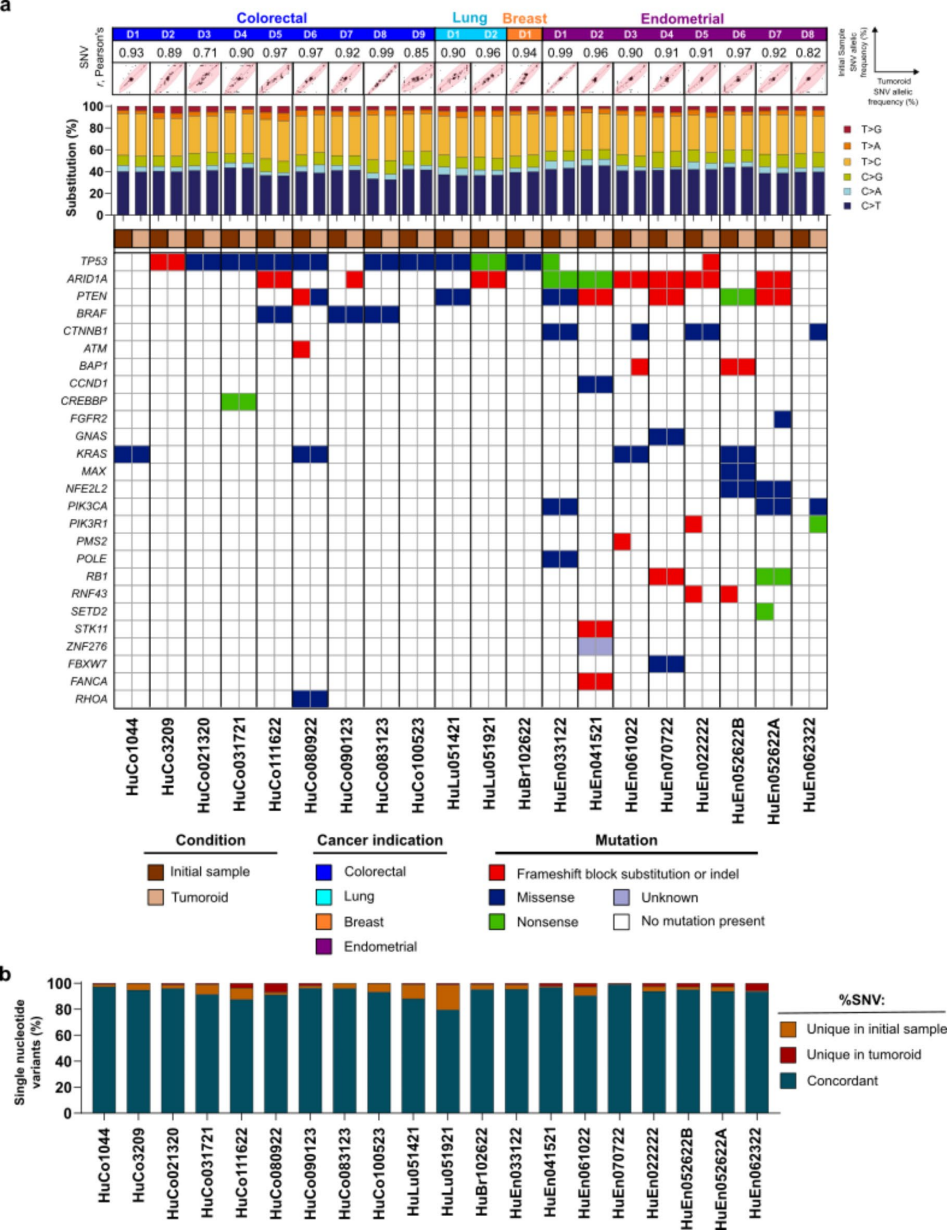
Tumoroids preserve donor-specific mutations from originating material. (**a**) Correlation of the allelic frequency of single nucleotide variants (SNVs; indicated by points) for matched tumor tissue and tumoroid pairs with regression line, confidence interval, and density ellipse encompassing 90% of the data (top), bar plots of single nucleotide base substitutions (middle), and oncogenic single nucleotide variants (bottom) for donor (D)-matched tumor (initial sample; brown box)/tumoroid (tan box) sets for the indicated cancer types and sample names (e.g., HuCo1044). In top panel, each dot represents the variant allelic frequency (VAF) for 1 genetic locus covered by the Oncomine Comprehensive Assay v3 (OCAv3). The oncogenic mutations were called using the Oncomine Variants filter 5.18 within the Ion Reporter software. Figure legend indicates cancer indication and mutation type detected. (**b**) Overlap in SNVs detected between initial donor material and established tumoroid lines (concordant SNVs, dark blue), and SNVs uniquely detected in each sample (orange, red) on a per-donor basis.

To help ensure that tumoroids preserve key genomic features of donor cancer material, we performed genomic characterization of successfully derived tumoroid cultures after 5–13 passages using the Ion Torrent Oncomine Comprehensive Assay v3 (OCAv3) for comparison to initial samples. Quantification of the variant allelic frequency (VAF) of single nucleotide variants (SNVs) detected by the assay revealed high concordance between matched tumors and tumoroids (Pearson correlation coefficient *r* ≥ 0.85) for colorectal (8/9), lung (2/2), breast (1/1), and endometrial (7/8) cancers (Fig. 2a, top panel). SNV profiles were patient-specific, with little cross-correlation across donors (*r* ≤ 0.70 between donors when all compared; see Supplementary Table S3 and Methods). The proportion of single base substitutions was also retained when comparing original tumor samples to corresponding tumoroids (Fig. 2a, middle panel). Furthermore, oncogenic driver mutations present in the original tumor samples were largely conserved in matched tumoroid cultures (Fig. 2a, bottom panel). On a per-mutation basis, > 87% of SNVs were shared between the initial samples and the derived tumoroids for 19/20 samples, suggesting minimal clonal loss or expansion in culture (Fig. 2b, Supplementary Table S4). The *APC* gene is commonly mutated in colorectal cancers^17^; as this gene is not covered by the OCAv3, we verified the presence of APC mutations in select colorectal tumoroids using quantitative polymerase chain reaction (qPCR) genotyping assays (Supplementary Table S5). Overall, these results indicate that the tumoroid media system can support samples across broad genomic landscapes from multiple cancer indications.

### Donor-specific transcriptomic profiles are preserved in tumoroid cultures

**Fig. 3:**
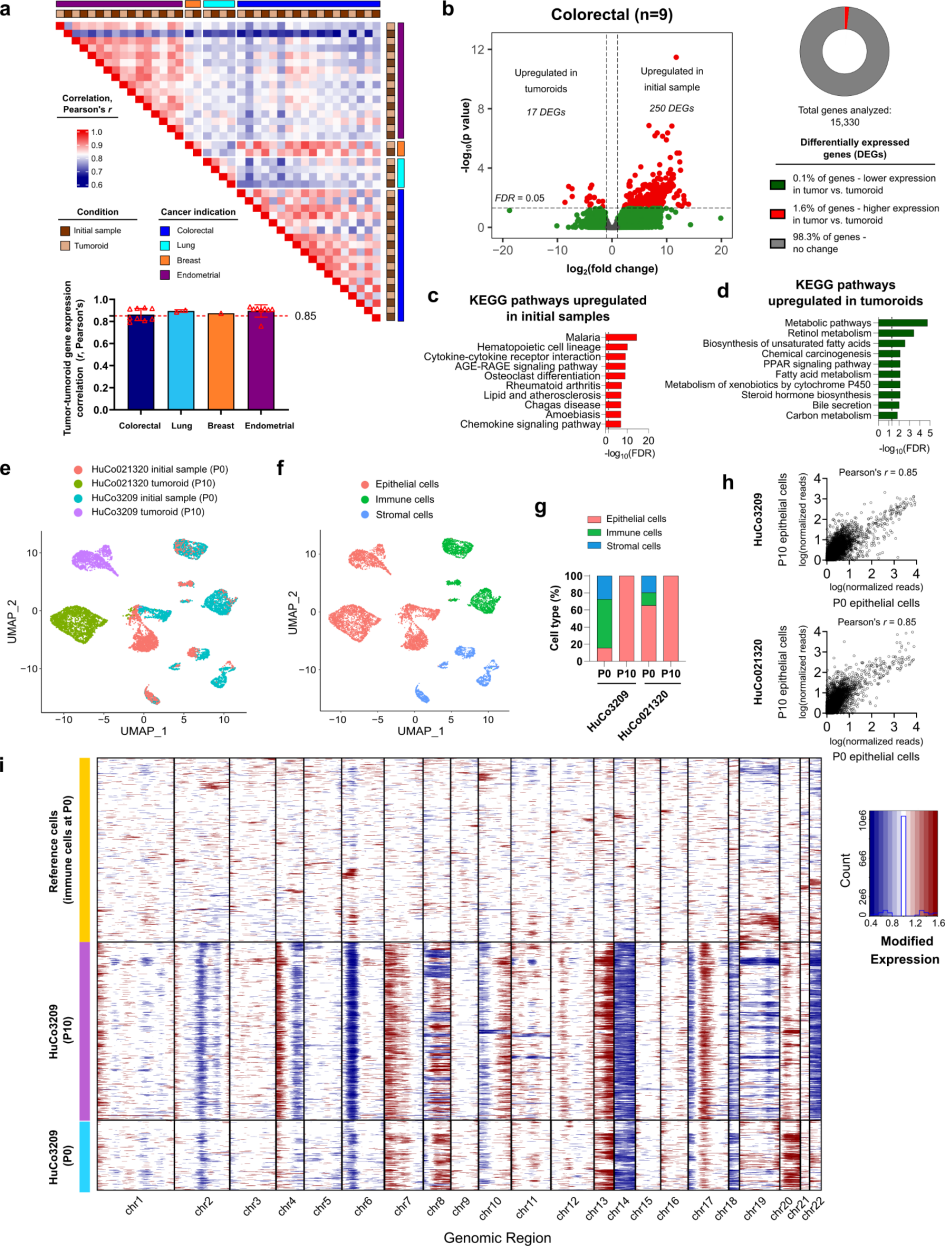
Tumoroids preserve transcriptomic features of original tumors. (**a**) Correlation heatmap of bulk RNA expression levels (log_2_(RPM + 1) levels correlated across 17,556 genes) in matched tumor tissue and tumoroids for the 20 colorectal, lung, breast, and endometrial cancer samples and tumoroids introduced in Fig. 2. Inset shows average (bar, mean ± standard deviation) Pearson’s correlation for matched tumor tissue and tumoroid pairs (dots denote unique donors) across different cancer indications. (**b**) Differential gene expression analysis in matched colorectal tumor tissue and tumoroids (*n* = 9) at fold change > 2 and false discovery rate (FDR) < 0.05. Samples analyzed are the 9 colorectal tumor/tumoroid sets shown in Fig. 2. (**c**) Top 10 significantly enriched Kyoto Encyclopedia of Genes and Genomes (KEGG) pathways of highly expressed genes in initial 9 biologically independent colorectal cancer samples (dotted line indicates FDR = 0.05). (**d**) Top 10 significantly enriched KEGG pathways associated with genes highly expressed in 9 derived colorectal tumoroid lines (dotted line indicates FDR = 0.05). (**e**) Uniform Manifold Approximation and Projection (UMAP) for dimension reduction visualization of 10,139 single cells from two matched tumor tissue/tumoroid pairs with color coded assignment of samples after single-cell RNA sequencing (scRNA-seq) for colorectal samples HuCo021320 and HuCo3209. (**f**) UMAP visualization with color-coded assignment of cancer cells, stromal cells, and immune cells. (**g**) Proportion of cell types present in initial samples (P0) and matched tumoroids (P10) for two colorectal cancer donors, HuCo021320 and HuCo3209. (**h**) Comparison of normalized, natural log transformed gene expression levels from pseudo-bulked scRNA-seq data of epithelial cells present in initial (P0) and tumoroid (P10) cells for HuCo3209 colorectal cells (17,731 genes) and HuCo021320 colorectal cells (17,536 genes). (**i**) Single cell copy number variation (CNV) plots comparing immune cells, tumor epithelial cells, and tumoroids for HuCo3209 donor. InferCNV was used to estimate CNV in epithelial cells using immune cells as the reference.

To analyze how closely transcriptomic profiles observed in human cancer samples resembled those from donor-matched in vitro tumoroid models, bulk RNA profiling was performed using the Ion Torrent Ion AmpliSeq Transcriptome Human Gene Expression Kit to quantify expression levels of > 20,000 human reference sequence genes in tumor and tumoroid pairs. Average Pearson’s correlation values to compare log-transformed, normalized gene read levels between matched tumor and tumoroid pairs of *r* = 0.86 (colorectal, *n* = 9), *r* = 0.89 (lung, *n* = 2), and *r* = 0.87 (endometrial, *n* = 8) were observed, suggesting high transcriptomic similarity (Fig. 3a). Additionally, the derived breast cancer tumoroid line showed a gene expression level correlation of *r* = 0.87 between initial cells and derived tumoroids. Overall, transcriptomic similarity was highest within a given cancer indication compared to cross-indication comparisons (Fig. 3a).

Differential gene expression analysis of primary tumors and tumoroids after expansion to establish early cryobanks (passage 3–16, depending on growth rate and initial number of cells seeded) showed minimal changes in gene expression levels associated with tumoroid establishment. Comparison of original tumors and colorectal tumoroids (*n* = 9) showed 267 differentially expressed genes (DEGs) at fold change > 2 and false discovery rate (FDR) < 0.05, accounting for less than 2% of analyzed genes (Fig. 3b). Pathway analysis of DEGs highly expressed in tumors but downregulated in tumoroids revealed that the top 10 most enriched Kyoto Encyclopedia of Genes and Genomes (KEGG) pathways were primarily immune cell signaling pathways (Fig. 3c). Similarly, minimal transcriptome changes of 4.4% and 1.0% were observed when comparing initial tumor cells to established tumoroids in lung cancer (*n* = 2) and endometrial cancer (*n* = 8), respectively, and differences in gene expression were primarily related to immune cell-related signaling pathways (Supplementary Fig. S3). Likewise, an unbiased gene expression analysis to identify the top 50 variable genes in our tumor/tumoroid data set followed by hierarchical clustering illustrated a decrease in gene expression levels associated with immune and stromal cells (e.g., *CCL3*, *CCL4*, *COL1A2*, *COL1A3*) when comparing tumors and derived tumoroids (Supplementary Fig. S3). This analysis showed retention of expression levels associated with epithelial cancer cells (e.g., *CDH17*, *KRT20*, *CDX1*, *NOX1*, *ESR1*, *SCGB2A1*, *SCGB1D2*, *PAX8*; Supplementary Fig. S3). Flow cytometry comparing cells dissociated from a primary tumor to its matched tumoroid culture confirmed the enrichment of EpCAM-positive and CEACAM-positive cells and a reduction in CD45-positive and CD31-positive cells during tumoroid establishment (Supplementary Fig. S3). The dropout of immune cell signatures is expected in established tumoroid cultures, as OncoPro medium is intended to culture cancer cells and is not expected to support immune and stromal cells over the long term, and is consistent with flow cytometry data indicating that stromal, immune, and endothelial cells that may be present in initial dissociated tumor samples are lost during long-term in vitro culture. Few genes (< 0.25%) were differentially upregulated in tumoroids for all three cancer indications, and those genes were primarily related to metabolism signaling pathways (Fig. 3d; Supplementary Fig. S3).

To directly compare the transcriptome of cancer epithelial cells from tumors and tumoroids, we performed single-cell RNA sequencing (scRNA-seq) of two dissociated tumor cell samples, HuCo3209 and HuCo021320, and matched tumoroid banks at passage 10 (Fig. 3e, f). Cells from the original tumor material formed distinct clusters of epithelial, immune, and stromal cells (as defined by expression of canonical marker genes; Supplementary Fig. S4) that were often nearby or overlapping between the two donors (Fig. 3e, f), though the proportion of cell types varied by donor (Fig. 3g). Established tumoroid cells were nearly all epithelial in nature and clustered most closely with the epithelial cells found in the initial sample (Fig. 3e-g), indicating that immune and stromal cells present initially were not retained in culture over the 59 day (HuCo021320) to 117 day (HuCo3209) period between passage 0 (P0) and passage 10 (P10). To directly compare gene expression levels in tumoroids with those in the epithelial cells present initially, scRNA-seq data from the epithelial cell subpopulations in initial samples (P0) and in tumoroids (P10) were pseudo-bulked and compared. Log-transformed normalized read counts between these populations were highly correlated for both HuCo3209 and HuCo021320 samples (*r* = 0.85; Fig. 3h).

Additionally, we investigated if colorectal tumoroids represent the original tumor at the copy number level. Genomic copy number variations in single cells classified as epithelial in both the initial (P0) sample and in established tumoroids were estimated using the inferCNV package^18^, with immune cells from the original samples used as the reference cells during analysis (Fig. 3i, Supplementary Fig. S5). Epithelial cells in the P0 samples had distinct regions of copy number gain or loss, which were largely conserved in tumoroid cells (Fig. 3i, Supplementary Fig. S5). Gene expression levels for the subset of genes used in the inferCNV analysis were highly correlated between the initial epithelial subpopulation and the established tumoroids, with Pearson’s correlation coefficient values of *r* = 0.77 and *r* = 0.75 for HuCo3209 and HuCo021320, respectively (Supplementary Fig. S5). In support of these findings, copy number estimates for genes covered by targeted DNA sequencing showed high levels of correlation between initial tumors and tumoroid cultures at the bulk sequencing level (Supplementary Fig. S5).

### Long-term expansion of tumoroids in suspension culture

**Fig. 4:**
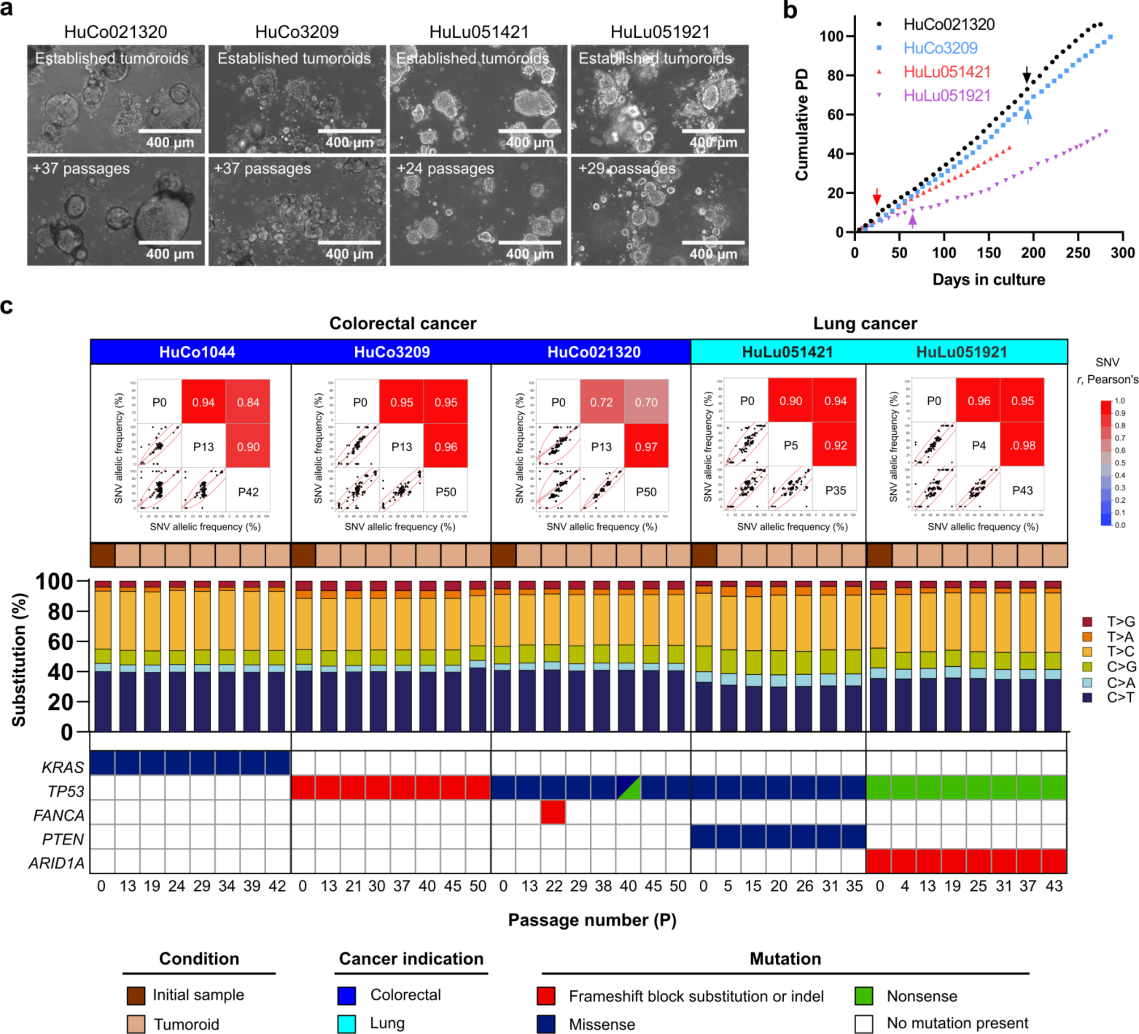
OncoPro medium-derived tumoroids are stable during long-term culture. (**a**) Morphology and (**b**) growth rate of tumoroid lines during long-term culture. Cumulative population doublings (PD) were measured by cell counts at each passage. Tumoroid lines were recovered from cryopreservation at the beginning of this experiment (day 0), and additional cryopreservation and recovery points are indicated by arrows for each culture. (**c**) Pearson’s correlation of single nucleotide variants (SNVs; indicated by points) between tumor samples (P0) and tumoroids at bank and at late passage numbers with regression lines, confidence intervals, and density ellipses encompassing 90% of the data (top). Each dot represents the variant allelic frequency (VAF) for 1 genetic locus covered by the Oncomine Comprehensive Assay v3 (OCAv3). Single base substitutions (bar graphs, middle) and oncogenic mutations (heat maps, bottom) from multiple time points during this study. The oncogenic mutations were called using the Oncomine Variants filter 5.18 within the Ion Reporter Software.

To evaluate the utility of patient-derived tumoroid cultures as long-term in vitro models, cryopreserved cultures derived from multiple colorectal and lung donors were thawed and maintained for up to 50 passages, with the cultures that were first established undergoing the longest evaluation. Experiments focused on suspension culture in non-tissue culture treated flasks with a low concentration of BME proteins (2% v/v) to provide ECM cues, a technique that facilitated routine maintenance of tumoroid lines. Patient-derived tumoroid cultures maintained their donor-specific morphologies during long-term suspension culture (Fig. 4a). Tumoroid density in these representative images should not be taken as an indicator of growth, as the cells floating in the suspension culture can drift. Instead, cell growth rate was calculated by dissociating tumoroids to single cells during passaging and reseeding a known number of cells. Cell doubling time was donor-dependent and averaged around 65 h for colorectal tumoroids – on par with that of 2D colorectal cancer cell lines – and 90–100 h for lung tumoroids. The established tumoroids grew steadily for over 40 population doublings (Fig. 4b). Testing included an additional cryopreservation and recovery step (indicated by arrows) and was concluded based on timing limitations, with no indication that cells would not expand further. Additionally, proof-of-concept experiments indicated that tumoroid lines expanded in suspension could be tumorigenic, with two of the lines tested (HuCo1044 and HuLu051921) forming tumors in NSG mice 40–80 days after subcutaneous injection of 3e6 cells/mouse. In both cases, resulting tumors showed histological similarity to the tumoroid cultures (Supplementary Fig. S6).

Genomic stability was assessed every 5–10 passages during suspension culture by characterizing patient-specific single nucleotide variants (SNVs) using targeted sequencing via the OCAv3 assay. For each culture, the allelic frequency of identified SNVs were compared between uncultured tumor cells (P0) and early- and late-passage cultures (Fig. 4c, top). Linear fits of this data show that SNV frequency is highly correlated (Pearson’s correlation coefficient) during long-term culture. In particular, high correlation coefficients (*r* ≥ 0.90) were observed between early and late passage cells, suggesting minimal drift over time in culture after tumoroid line establishment (Fig. 4c, top). To understand the generalizability of this finding, a tumoroid line (HuCo021320) was transferred to another site and expanded from cryopreservation for > 20 passages by different users. Similar correlation values were observed in SNVs (Supplementary Fig. S7). Further analysis of single base substitutions showed conserved patterns of substitution percentages and transition-transversion mutations between tumor tissue and matched tumoroid cultures during extended in vitro propagation (Fig. 4c, middle). Finally, known driver mutations were well conserved from tumor tissue across multiple passages in tumoroid culture (Fig. 4c, bottom), with uncommon instances of new mutations being called, some of which were not called again in sequencing runs of later passage cultures.

**Fig. 5:**
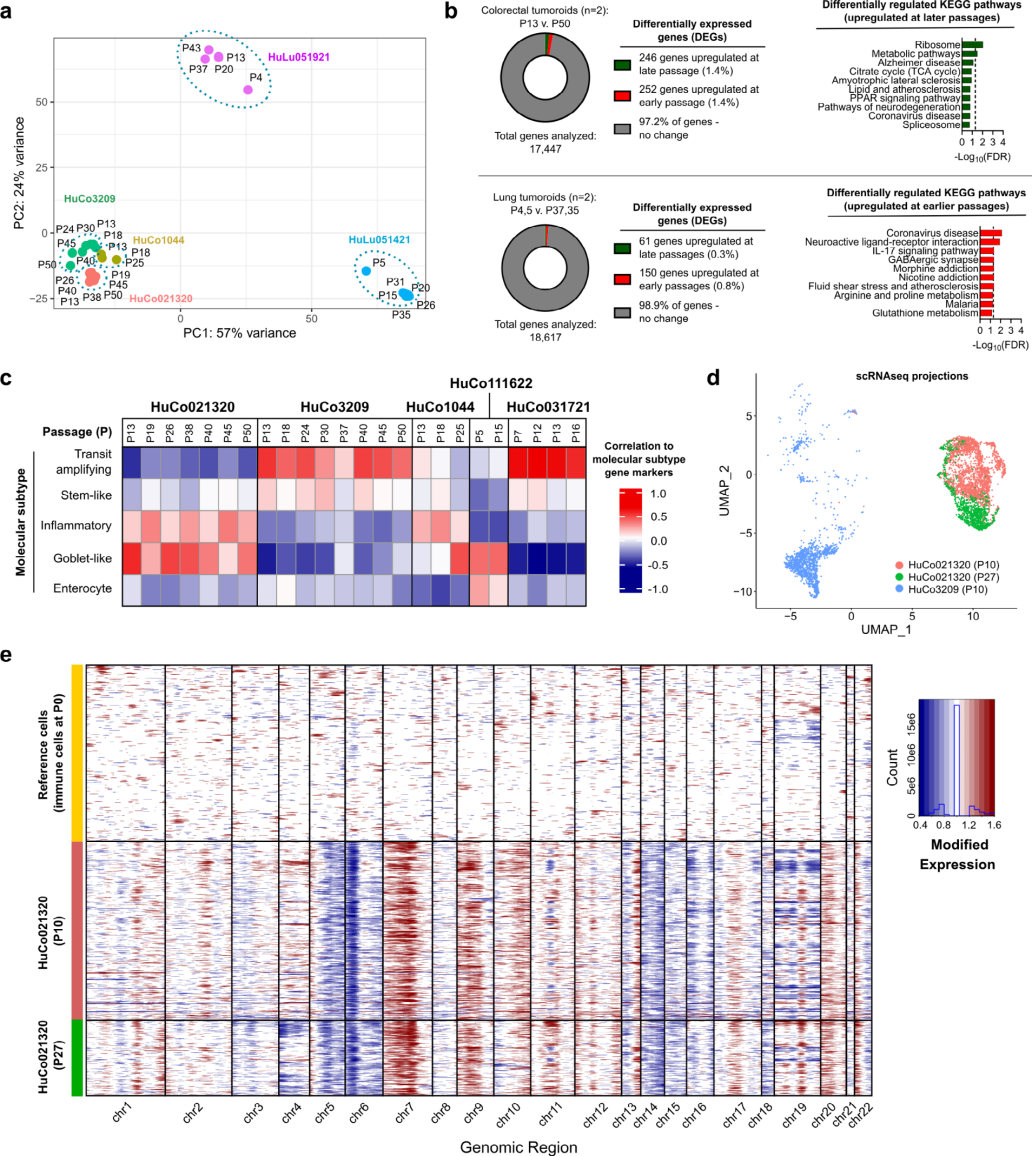
OncoPro medium-derived tumoroid transcriptomes are stable during long-term culture. (**a**) Principal component analysis (PCA) of global gene expression patterns in human colorectal (HuCo) and lung (HuLu) tumoroid cultures over multiple passages (P). (**b**) Differential gene expression (DEG) analysis of early- versus late-passage tumoroids, with DEGs called at fold change > 2 and false discovery rate (FDR) < 0.05, for HuCo and HuLu samples. Pathway analysis of DEGs revealed few significantly enriched Kyoto Encyclopedia of Genes and Genomes (KEGG) pathways (FDR < 0.05) in late-passage colorectal tumoroids and early-passage lung tumoroids; the top 10 KEGG pathways for colorectal and lung tumoroids are shown. No KEGG pathways were enriched in early-passage colorectal tumoroids or late-passage lung tumoroids. Dotted line indicates FDR = 0.05. (**c**) Consensus molecular subtypes from gene expression analysis of colorectal patient-derived tumoroids through multiple passages. (**d**) Uniform Manifold Approximation and Projection (UMAP) for dimension reduction plot of single-cell RNA sequencing data from 4,905 total cells for HuCo021320 (early, P10; and late, P27) and HuCo3209 (early, P10) tumoroid cultures. HuCo3209 was used as a control donor for comparison. (**e**) Single cell copy number variation (CNV) plots for HuCo021320 (*n* = 4,973 genes, by column) of early (P10) and late (P27) passage tumoroid cultures. Each row represents one cell. InferCNV was used to estimate CNV in epithelial cells, using immune cells present in dissociated tumor tissue (P0) as reference.

Transcriptome stability in patient-derived tumoroid cultures was first assessed using bulk RNA sequencing. Samples from multiple passages clustered by donor during principal component analysis (PCA; Fig. 5a), and gene expression levels clustered by donor in unsupervised hierarchical clustering analysis of Euclidean distances between the samples (Supplementary Fig. S8). Similarly, tumoroid growth rates and gene expression levels were conserved for a given donor across different medium production lots (Supplementary Fig. S7). Differential gene expression analysis of 2 colorectal tumoroid donors at early (P13) vs. late (P50) timepoints demonstrated no significant change in > 97% of genes (Fig. 5b). Pathway analysis of differentially expressed genes (DEGs) in either early- or late-passage colorectal tumoroids revealed two significantly enriched KEGG pathways relating to protein translation and metabolism in late-passage tumoroids, with no KEGG pathways relatively enriched in early-passage tumoroids (Fig. 5b). Similarly, comparison of early passage (P4 or P5) to late passage (P37 or P35, respectively) lung tumoroids revealed that only 1.1% of genes were differentially expressed, with enrichment for a small number of KEGG pathways primarily related to inflammatory and neural signaling in early-passage tumoroids and no significantly upregulated pathways at later passages (Fig. 5b). Having demonstrated the maintenance of overall gene expression patterns during long-term culture, we next asked if the clinical subtype of tumoroids was maintained. We used a gene expression-based PAM38 classifier^19^ to classify colorectal cancer tumoroids into 5 consensus molecular subtypes: enterocyte, goblet-like, inflammatory, stem-like, and transit amplifying. Tumoroids from different donors represented unique subtypes (Fig. 5c), indicating that diverse phenotypes can be maintained in tumoroid culture. HuCo021320 tumoroids resembled goblet and inflammatory subtypes; HuCo03209 and HuCo031721 tumoroids most closely matched the transit amplifying subtype, with a small proportion of stem-like cells; HuCo111622 cells were mostly enterocyte or goblet-like; and HuCo1044 tumoroids contained mixed subtypes (Fig. 5c). Importantly, all the donors used in this analysis represented their respective clinical subtypes when cultured for 10–37 passages. Characterization of HuCo021320 colorectal cancer tumoroids by scRNA-seq showed that passage 27 tumoroids overlapped with passage 10 tumoroid samples and were distinct from those from another donor (Fig. 5d), demonstrating maintenance of identity at the single-cell level. Additionally, CNV estimates inferred from scRNA-seq data (Fig. 5e) or estimated from targeted genomic sequencing (Supplementary Fig. S9) indicated that tumor-specific ploidy values were maintained from early- to late-passage tumoroids.

### Growth and genotypic stability of previously established tumoroid cultures in OncoPro medium

**Fig. 6:**
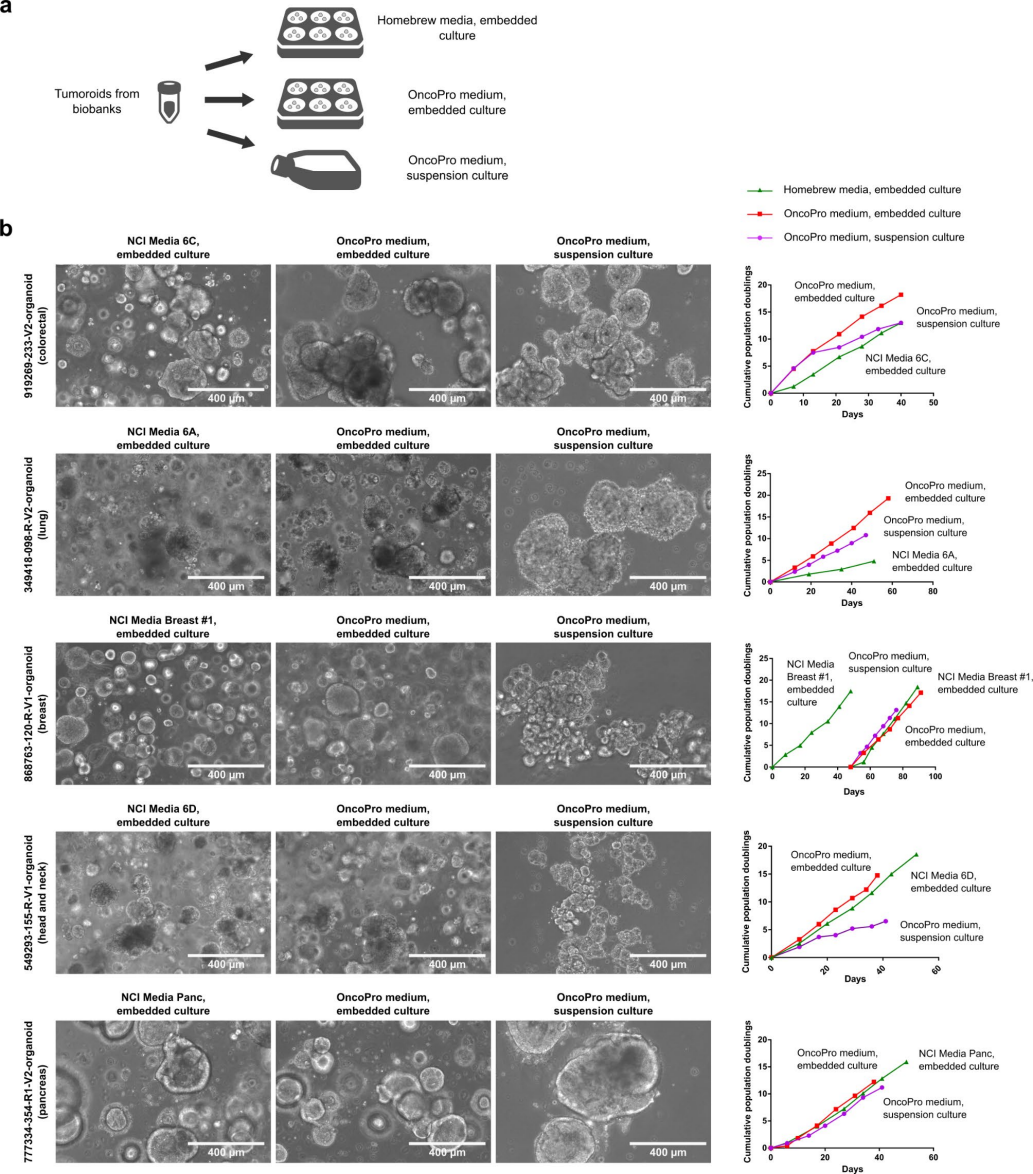
Publicly available tumoroid lines can adapt well to culture in OncoPro medium. (**a**) Outline of study comparing growth rate of tumoroids from biobanks (for example, National Cancer Institute Patient-Derived Models Repository, PDMR) in homebrew medium to growth of tumoroids in OncoPro medium in embedded or suspension culture formats. (**b**) Tumoroid lines from colorectal, lung, breast, head and neck, and pancreas cancer cultured in PDMR-recommended homebrew media or in OncoPro medium in embedded or suspension culture have similar morphologies and growth rates. For model 868763-120-R-V1-organoid, cells were initially expanded in PDMR-recommended media for several passages to create a working bank and then cultured in all three conditions in parallel. Scale bar = 400 μm.

To test the generalizability of the culture system with publicly available tumoroid models, we next tested the expansion of tumoroid cells from the United States National Cancer Institute Patient-Derived Models Repository (NCI PDMR)^20^, and an additional, commercially available colorectal tumoroid line (3dGRO ISO72, Millipore Sigma). These models come with recommendations for use of culture media that is generated by combining basal medium with various supplements, growth factors, and, at times, conditioned media, which we collectively refer to as “homebrew” tumoroid media. Models tested were procured as cryopreserved cells. In our experiments, cells were plated into BME-embedded culture in source-recommended media (NCI PDMR recommended homebrew media or vendor-recommended homebrew medium; see Materials and Methods and Supplementary Table S6 for details and modification of NCI PDMR media recipes to incorporate recombinant proteins), or in OncoPro medium in either embedded or suspension formats (with indication-specific growth factors and supplements as required; see Supplementary Table S1) in parallel (Fig. 6A). Specifically, OncoPro medium was supplemented with 10 ng/ml HS FGF-10 for head and neck tumoroid cultures and with 10 ng/ml HS FGF-10 plus 10 nM gastrin I for pancreas tumoroid cultures; colorectal, lung, and breast tumoroids were cultured as described above. Testing was performed either upon receipt of cryopreserved material or after expansion of a tumoroid bank from initial NCI PDMR material using modified NCI PDMR media recipes (see details in Supplementary Table S6 and Supplementary Table S7). Growth of cells was monitored over time by obtaining cell counts at each passage, reseeding a known number of cells, and calculating cumulative population doublings. Additionally, cells were sampled for DNA and RNA purification from each condition upon culture initiation and after 3–6 passages post-thaw for most cultures (see Supplementary Table S7). In some instances, low initial cell yield precluded the sampling of cells at the time of initial thaw for RNA sequencing. Testing was performed using 4 colorectal tumoroid lines, 4 lung tumoroid lines, 4 breast tumoroid lines, 3 head and neck tumoroid lines, and 3 pancreas tumoroid lines (Supplementary Table S7). No crypt villus organoids or colon crypt organoids were observed as only carcinoma cells were procured for this study.

In our testing, morphologies and growth rates of tumoroid cultures were similar across media systems and culture methods (Fig. 6b; Supplementary Fig. S10). In some cases, tumoroid growth rate was higher in embedded culture than in suspension culture, and vice versa. Growth rates were generally similar between the NCI PDMR or vendor recommended medium and OncoPro medium, with some models exhibiting a slight growth advantage in one system over the other. For some tumoroid lines, cells from one arm of the study were lost during culture or were not fully tested in the suspension culture format (denoted as “Data not available”; see also Supplementary Table S7). One NCI PDMR lung cancer line, LG0481, did not expand in OncoPro medium but grew in NCI PDMR Media 6A, while a head and neck cancer tumoroid line, 832693-133-R-V1-organoid, expanded in OncoPro medium in embedded and suspension formats but not in the recommended NCI PDMR Media 6D (Supplementary Fig. S10).

**Fig. 7:**
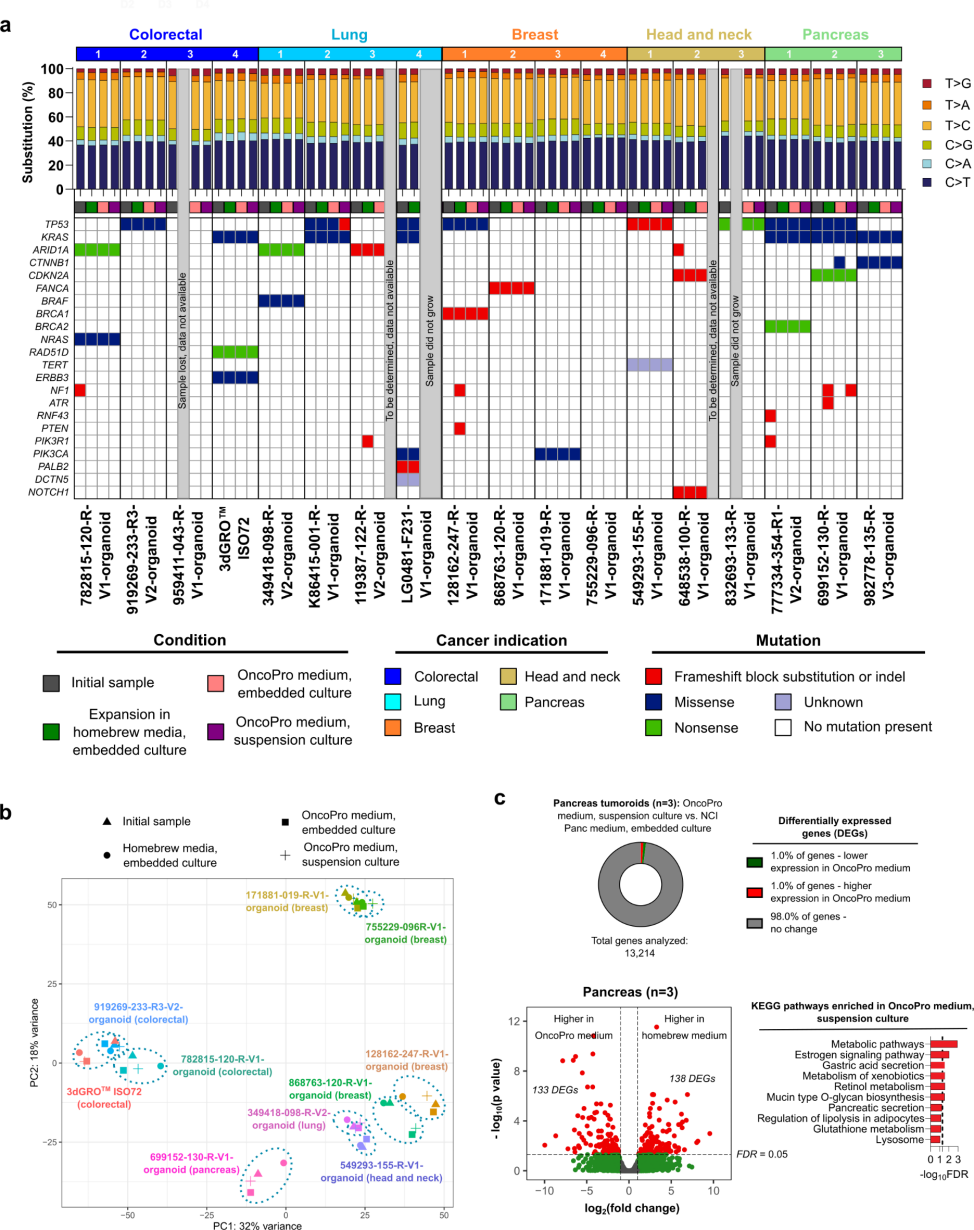
Previously established tumoroid lines adapted to OncoPro medium have similar genomic and transcriptomic characteristics compared to the cultures grown in homebrew media. (**a**) Single nucleotide base substitutions (top) and oncogenic single nucleotide variants present in the samples following sequencing with the Oncomine Comprehensive Assay v3 (OCAv3) and mutation calls called using the Oncomine Variants filter 5.18 within the Ion Reporter software (bottom). (**b**) Principal component analysis (PCA) plot comparing gene expression levels in initial cells (triangles; directly from vendors or from internal cell banks) and cells expanded using homebrew embedded culture (circles), OncoPro medium embedded culture (squares), and OncoPro medium suspension culture (crosses). (**c**) Differential gene expression analysis of pancreas tumoroids cultured in PDMR-recommended homebrew media (embedded format) and OncoPro medium (suspension format; *n* = 3), showing differentially expressed genes (DEGs) at fold change > 2 and false discovery rate (FDR) < 0.05. Percentage change in transcriptome (**c**, top) and analysis for enriched Kyoto Encyclopedia of Genes and Genomes (KEGG) pathways in genes highly expressed during suspension culture in OncoPro medium (**c**, bottom). Top 10 results are shown. Dotted line indicates FDR = 0.05. No KEGG pathway was enriched when highly expressed genes in homebrew medium were used in the analysis.

To test whether a change in media system or culture format was associated with genetic or transcriptional drift in these models, we performed targeted mutational profiling and transcriptional analysis to compare initial cells (as received, or cells from an internally established cell bank using NCI PDMR media; see Supplementary Table S7 for details) to cells expanded in various arms of the study. Tumoroid cultures from different donors and cancer indications harbored a heterogeneous set of cancer driver genes affected by missense mutations, non-sense mutations, or frameshift mutations (Fig. 7a). In general, oncogenic mutations were preserved between culture conditions, though some mutations were detected in initial material that were not called in one or more expansion arms, and vice versa (Fig. 7a). No clear pattern of loss or emergence of mutations following culture in a given media system or culture format was noted, and SNV presence and variant allelic frequency were highly concordant for a given tumoroid line (*r* > 0.97 when all samples were batched and compared) regardless of the system in which they were grown (Fig. 7a, Supplementary Table S8). The minimal cross-culture correlations observed in this data indicate retention of donor specificity (Supplementary Table S8). SNV concordance between initial cells and tumoroids expanded in each arm of the study was also high (> 97% for 47/48 of comparisons), indicating that culture conditions were not driving the loss or emergence of SNVs (Supplementary Fig. S11, Supplementary Table S9).

PCA of gene expression profiles from initial samples (received from vendor or from initial cell bank) versus those expanded in this study showed clustering by donor rather than by growth condition, indicating maintenance of gene expression profiles in OncoPro medium (Fig. 7B). In some instances, low initial cell yield precluded the sampling of cells at the time of initial thaw for RNA sequencing; including samples in which we could not obtain an initial gene expression profile led to similar clustering by PCA after bulk RNA sequencing (Supplementary Fig. S12). To understand what differences are driven by the suspension format, we compared 15 pairs of suspension and embedded tumoroid cultures (*n* = 3 colorectal, *n* = 4 lung, *n* = 2 pancreas, *n* = 2 head and neck, *n* = 4 breast) maintained in OncoPro medium at similar passage numbers. In this comparison, only 1% of genes were differentially expressed (Supplementary Fig. S12). Genes upregulated in embedded culture were related to stress and hypoxic response, possibly due to the relatively higher cell-cell proximity within these cultures compared to the suspension format.

On an indication-specific basis, tumoroids expanded in OncoPro medium suspension culture and in homebrew media embedded culture were further compared to elucidate differences driven by the suspension culture approach. For pancreas cancer samples, only 2% of genes were differentially expressed between these conditions (Fig. 7C). KEGG analysis showed that genes highly expressed during suspension culture in OncoPro medium (compared to embedded culture in NCI Panc medium) were associated primarily with metabolic signaling pathways (Fig. 7C). No KEGG signaling pathways were enriched when analyzing genes upregulated in NCI Panc medium. Similar results were obtained for colorectal, lung, and breast cancer tumoroids, where expression levels for < 4% of genes were significantly different when comparing tumoroids expanded in homebrew embedded culture to those grown in OncoPro medium in the suspension format (Supplementary Fig. S13).

Clinical subtypes based on gene expression levels were also generally retained across media systems. Classification of colorectal cancer tumoroids using the PAM38 gene classifier showed that original and OncoPro medium cultures of the 782815-120-R-V1-organoid, 919269-233-R3-V2-organoid, and 3dGRO ISO72 tumoroids maintained the transit amplifying, goblet-like/inflammatory, and transit amplifying/stem-like subtypes present in the original samples, respectively, after culture in OncoPro medium (Supplementary Fig. S13). Similarly, the basal subtype of triple negative 128162-247-R-V1-organoid and 868763-120-R-V1 breast cancer tumoroids (by the PAM50 gene signature) was consistent across culture formats (Supplementary Fig. S13). However, PAM50 subtyping of 171881-019-R-V1-organoid and 755229-096-R-V1 lines showed that OncoPro cultures adopted HER2 subtypes, while the original cultures had characteristics of luminal B cancers. These lines are positive for estrogen receptor (ER) and progesterone receptor (PR) expression (ER+/PR+), and we noted that the expression of estrogen receptor 1 (ESR1) and progesterone receptor (PGR) genes was lower in these cultures after transition to OncoPro medium. Of note, the expression of these genes was still significantly higher in the OncoPro medium-transitioned ER^+^/PR^+^ lines when compared to the triple-negative lines, indicating retention of ER^+^/PR^+^ expression in OncoPro medium (Supplementary Fig. S13). Other groups have shown some loss of these receptors in 30% of conditions^21^, and further optimization of medium conditions may be required for maintenance of ER/PR levels that more consistently reflect those observed in clinical samples. Overall, these data indicate that OncoPro medium can be used for the culture of existing tumoroid models from colorectal, lung, breast, head and neck, and pancreas cancers, with minimal expected changes in growth rate, mutational profile, or gene expression levels.

## Tumoroid suspension cultures are amenable to medium-throughput, multiplexed immune cell-mediated cytotoxicity assays

**Fig. 8:**
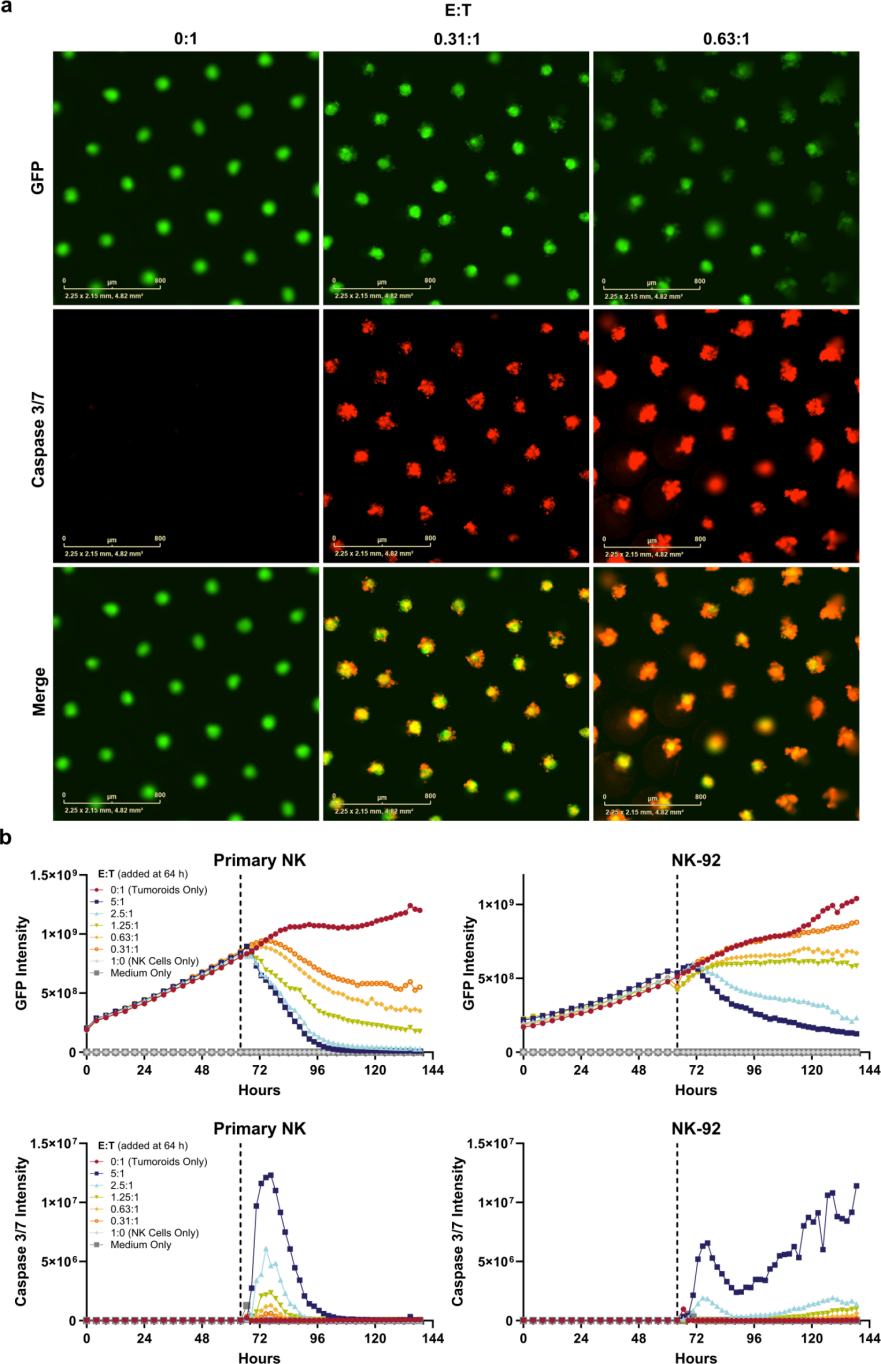
Tumoroid suspension cultures are amenable to multiplexed assays for immune cell cytotoxicity studies. A colorectal tumoroid line (HuCo1044) stably expressing green fluorescent protein (GFP) was dissociated and seeded into 96 well microcavity plates. After 64 h, natural killer (NK) cells from a primary source or the NK-92 cell line were added at various effector to target (E: T) ratios along with an indicator of caspase 3/7 activity, Invitrogen CellEvent Caspase-3/7 Red. (**a**) Representative images of HuCo1044-GFP cells acquired on an Incucyte Live-Cell Analysis System 24 h after addition of primary NK cells at multiple E: T ratios (0:1 represents tumoroids only condition). Scale bar = 800 μm. (**b**) Quantitative image analysis of green (HuCo1044-GFP) or red (caspase-3/7 activity) integrated fluorescence intensity (green or red calibrated units × µm^2^/well) before and after addition of Primary NK or NK-92 cells at 64 h (dotted line). Plots show average intensity values across three replicate wells per condition.

To demonstrate the compatibility of tumoroids with genome engineering and cytotoxicity assays, we engineered a colorectal tumoroid line, HuCo1044, to express green fluorescent protein (GFP). After dissociation and exposure to lentivirus in suspension culture, 67% of tumoroid cells were GFP-positive (GFP+), which increased to over 99% after antibiotic selection (Supplementary Fig. S14, Supplementary Video S1). Targeted genomic sequencing and transcriptome profiling indicated that the engineered cells had a high degree of similarity to the parental culture (Supplementary Fig. S14). To simulate immune-oncology development workflows, we co-cultured GFP + tumoroids with primary natural killer (NK) cells or an immortalized NK cell line, NK-92. Primary NK cells isolated from peripheral blood mononuclear cells via negative selection demonstrated 94.8% purity (CD45 + CD56+), were not enriched for CD3 + T cells (0.25%), and contained 33.3% CD16^bright^ cells (Supplementary Fig. S14). Tumoroids (target cells) were passaged and seeded alone, and their expansion could be tracked by GFP signal (Fig. 8a, b). After 64 h, NK cells (effector cells) were seeded at various effector to target (E: T) ratios. Co-incubation of tumoroids and NK cells led to a dose-dependent decrease in tumoroid GFP signal and a concomitant increase in caspase 3/7 activity (Fig. 8a, b). Quantitative image analysis demonstrated that tumoroid-only control cells continued to grow, leading to an increase in GFP signal in the absence of caspase activity, while the presence of NK cells led to decreasing GFP intensity in a dose-dependent manner (Fig. 8b). Similar results were demonstrated with the NK-92 cell line, though its killing efficiency appeared lower than that of the primary cells (Fig. 8b). In summary, tumoroids cultured in OncoPro medium were compatible with genetic engineering and could be utilized to demonstrate the effectiveness of cancer-targeting regimens.

## Discussion

Here, we demonstrate the utility of a novel tumoroid culture medium, Gibco OncoPro Tumoroid Culture Medium, for the culture of patient-derived 3D tumoroid models. The system was used for both (1) deriving novel tumoroid lines from cryopreserved dissociated tumor cells and fresh surgical resection samples, and (2) culturing established cancer organoid lines. When supplemented with tissue-specific niche factors, we used OncoPro medium for culturing tumoroids from a variety of solid tumors, including colorectal, lung, pancreatic, head and neck, endometrial, and breast cancers. During culture, mutations and gene expression signatures characteristic of the starting material were retained (Figs. 2, 3 and 7), and select lines tested were tumorigenic (Supplementary Fig. S6). Furthermore, tumoroids from a wide range of tumor stages, grades, mutational backgrounds, molecular subtypes, and donor demographics were supported (Figs. 2 and 7; Supplementary Table S2). There is not a clear pattern of these variables that determines compatibility with the medium, though this bears monitoring as sample size increases. Additionally, the genetic and transcriptomic concordance of derived tumoroids with the starting material was not affected by whether fresh tissue or cryopreserved dissociated tumor cells were used to initiate cultures for a limited number of colorectal cancer samples tested (Figs. 2 and 3; Supplementary Figures S3-S5; Supplementary Tables S3-S4; list of whether tumoroids were initiated from fresh tissue or cryopreserved dissociated tumor cells is given in Supplementary Table S2). Future studies to quantify the effects of tissue handling prior to initiating tumoroid culture are warranted. We expect that supporting niche factors that enable culture from additional cancer indications will be uncovered, and additional efforts using both fresh tissue resections and existing tumoroid models from the NCI PDMR are in progress.

Tumoroid culture establishment represents a key technology for enabling patient-derived cancer models and is more affordable than establishing novel patient-derived xenograft (PDX) models^22^. Previous studies suggest that tumoroid and PDX models retain donor-specific mutations, histological markers, and gene expression levels from the originating tumor to a similar degree and exhibit generally concordant responses to drugs^23–25^. Direct comparison of a PDX model, tumoroid model, and 2D cell lines derived from an ovarian clear cell carcinoma patient demonstrated that the PDX and tumoroid models recapitulated patient response to treatments, while the 2D cell line model did not^26^. In our studies, the definition of a stable tumoroid line was one that: exceeded 5 cumulative population doublings based on the number of cells initially seeded in culture, was passaged every 14 days or less for over five passages, was freeze/thaw competent, and was genetically and transcriptionally similar to the initial tumor material (SNV VAF correlation *r* > 0.7, tumor-tumoroid gene expression correlation *r* > 0.7). The success rate for stable tumoroid line establishment varied depending on multiple factors, such as time between tumor excision and culture and whether dissociated cells had been cryopreserved, but, with an optimized protocol, remained around 50% for colorectal and endometrial cancers. Our studies with a limited number of cryopreserved, dissociated colorectal tumor cell samples show that while culture initiation from these sources is feasible, the time to reach 5 cumulative population doublings from the initially plated cell number may be longer when cryopreserved samples are used (Fig. 1c and source data). More study will be required to understand the impact of using fresh vs. cryopreserved cells on derivation success rate. The endometrial cancer data sample set we studied during focused derivation experiments (which excludes model HuEn041521) constituted 15 fresh tumor samples that were received and processed within 24 h of tumor resection. Of those samples, 14/15 initially formed tumoroids in culture, 11/15 samples reformed tumoroids for several passages (passaging every 1–2 weeks), and 7/15 samples were able to grow stably for as long as they were tested (100–200 days). Our initial studies suggest that this success rate is lower in lung and breast cancers. In general, we noted much higher success rate (> 85%) of tumoroid formation during short-term culture across indications, as also observed by others^27,28^, making short-term studies feasible for a wider range of samples. Unsuccessful cultures failed to re-form 3D tumoroid structures after dissociation (sometimes after several passages) or stopped expanding in vitro, precluding their use as stable, expandable cell models. Additional protocol improvements or tissue-niche factors may further enhance derivation rates; others have demonstrated that derivation using multiple bespoke homebrew media in parallel can further enhance success^29^.

Altogether, tumoroids retain the major genomic mutations of the tumors from which they were derived. Targeted genomic profiling to compare tumor material to matched tumoroid cells revealed occurrence of oncogenic mutations and donor-specific SNVs at similar allelic frequencies in both sets. Additionally, we observed qualitatively similar base substitution patterns between initial tumor samples and established tumoroids in donor-matched comparisons (Fig. 2), with T > C and C > T as the most abundant base substitutions in all samples, consistent with other findings^30,31^; indeed, T > C and C > T substitutions have been associated with loss of heterozygosity and gain of heterozygosity across multiple cancer types^32^. We noted some donor-to-donor variability in substitution patterns (for example, relatively fewer C > A substitutions in HuEn070722 compared to other endometrial samples) within the largely similar substitution patterns for a given donor. Copy number variations were also maintained, as estimated broadly over the genome using scRNA-seq and inferCNV or by the OCAv3 assay. Mutations identified in tumor and tumoroid samples will depend on the sequencing method and analysis pipeline, and broader genomic sequencing would supplement the targeted approach utilized here. Comparison of epigenetic modifications to tumor cells would also be worthwhile given recent findings that tumoroids retain greater sensitivity to epigenetic vulnerabilities than 2D cell lines^33^.

The loss of expression of immune and other stromal cell signaling pathways during derivation in OncoPro medium represented the major changes in the gene expression profiles of tumoroid cultures when compared to primary tumor material (Fig. 3). In concordance with this finding, flow cytometry and single cell RNAseq data demonstrate that stromal and immune cells present in the initial tumor sample are not supported over long-term culture in OncoPro medium (Fig. 3, Supplementary Fig. S3,S4). KEGG pathway analysis of genes upregulated in initial colorectal tumor samples compared to established colorectal cancer tumoroids identified pathways that are likely associated with paracrine signaling between cancer cells and cells in the microenvironment, such as hematopoietic cells (Fig. 3c); this signaling appears to be lost when non-epithelial cells are not retained during long-term tumoroid culture. Other media systems have also demonstrated loss of signaling associated with non-malignant cell components over the course of weeks in culture^27^. At the single-cell level, comparison of malignant tumor cells and tumoroid cells demonstrated that transcriptomes were similar, with a correlation coefficient of 0.85, further indicating that loss of non-tumor cell populations drive much of the observed transcriptional changes observed during culture establishment. Transcriptional changes within the tumor cell population may be caused by removal of cell-cell contacts or paracrine signaling from non-malignant cells. Preservation or re-addition of other cell types in co-culture or using in vivo models may restore these cues^34,35^. Given the importance of immune, stromal, and vascular cells present in the tumor microenvironment to treatment response and resistance^36–39^, future work should explore preservation of non-epithelial cells in tumoroids or addition of these cells to tumoroid models when such model complexity is necessary^40^. The lack of these cells is a shortcoming of our current model and is one area, along with metabolic processes, where initial tumor samples may resemble PDX models more closely than tumoroids^23^.

As compared to several other published methods^11,13,21,41–43^, our proprietary and novel serum-free medium does not require conditioned medium or Wnt agonists such as R-spondins. Additionally, no small molecule inhibitors are present in the OncoPro basal medium, OncoPro Supplement, BSA supplement, or B-27 supplement. The lack of Wnt activation may protect against non-malignant cells outcompeting malignant cells in tumoroid line derivation, a known issue with some cancer indications, including lung and breast cancers^14,16,44^. Despite lacking Wnt agonists, OncoPro medium supported multiple tumoroid models from the NCI PDMR that are typically grown in systems containing Wnt and R-spondins. This is in line with literature suggesting that exogenous Wnt activation may not be required for in vitro growth of multiple cancer indications^29,45,46^, either by gene mutation, endogenous production, or through transcriptional programming. In line with this, we verified the presence of APC mutations^17^ in select colorectal tumoroids (Supplementary Table S5). In addition to Wnt agonists, other factors and small molecules demonstrated to be necessary for the growth of non-malignant organoids are present in many published tumoroid media formulations^21,43,45^. The absence of Wnt agonists, R-spondins, noggin, and small molecule inhibitors (prior to the addition of Y27632 in some culture protocols) in the OncoPro medium formulation makes this system incompatible with the long-term culture of adult stem cell-derived organoids. Organoids derived from non-diseased tissue are used for comparison to tumoroids in some assays, and while our system prevents overgrowth by non-malignant cells in culture, one drawback is that a separate media system would be required for experiments incorporating healthy adult stem cell-derived organoids.

Once established, comparison of early and late passage tumoroid cultures indicated stable growth rates, with retention of CNV levels and SNV presence and frequency, demonstrating stability over months in culture. This result held for both internally-derived tumoroid lines and tumoroids procured from external parties, where OncoPro medium supported commercially available tumoroid models (17/18 tested here). Testing revealed the importance of beginning with sufficient cell number from high-quality cryobanks or ongoing cultures, and passage of individual cultures when tumoroids reached sufficient size to prevent overgrowth (even if it meant passaging different media conditions at different times). Overall, growth and morphology were similar between homebrew media systems and OncoPro medium embedded or suspension cultures, with occasional donor-to-donor variability in growth rate between culture formats or media. One lung adenocarcinoma line (LG0481-F231-V1-organoid) expanded in the NCI recommended medium but not in OncoPro medium, while one lip/oral cavity squamous cell carcinoma (head and neck) sample (832693-133-R-V1-organoid) expanded in OncoPro medium but not the NCI recommended medium in our hands. NCI PDMR standard protocols rely on the addition of fetal bovine serum (FBS) through addition of L-WRN conditioned medium, and it is possible that the instances in which tumoroid growth was slower in NCI PDMR media compared to OncoPro medium was influenced by our approach using recombinant Wnt (W), RSPO (R), and noggin (N) sources, which changed the source of WRN in the media and eliminated FBS from these cultures. While we believe that the reproducibility and ease-of-use of the OncoPro medium system offers substantial advantages to homebrew approaches, 17 of the 18 NCI PDMR tumoroid models that we tested also expanded in homebrew media (Fig. 6, Supplementary Figure S10, Supplementary Table S6), which remains an alternative option to culture tumoroid models. Additional homebrew media recipes have been used and described for tumoroid derivation and culture in the literature^10,11,13,21,25,27,29,35,41–43,45–52^, and these could also serve as potential alternatives to OncoPro medium.

Critically, genomic mutations and gene expression patterns were maintained during culture of established tumoroid lines in OncoPro medium in both embedded and suspension formats. These comparisons also revealed that few genes were differentially expressed after months in culture. Molecular subtypes of colorectal and triple-negative breast cancers were maintained during tumoroid culture, suggesting that key clinical aspects of gene expression signatures are stable during culture using OncoPro medium. Such maintenance of clinical subtype during tumoroid establishment has been named as critically important to demonstrate the physiological relevance of in vitro cancer models, especially with regard to drug response^21,47,53^, and previous publications have mixed claims regarding the ability of tumoroids to adequately maintain subtypes representative of the tissue from which the tumoroid was derived or generate biobanks with tumoroid lines representing certain clinical subtypes. In line with this result, we observed some shift of hormone-receptor positive lines towards a HER2 subtype in OncoPro medium, with concomitant decreases in estrogen receptor and progesterone receptor gene expression levels compared to cryopreserved tumoroids received from the NCI PDMR (Supplementary Fig. S13). Molecular subtyping approaches are dependent on the timepoint for comparison (tissue versus early passage for later comparison), cancer type of interest, assay of choice (scRNA-seq versus bulk RNA sequencing), and analysis pipeline, to name a few, and can thus be difficult to compare across studies. Future testing of the functional impact of maintenance of molecular subtype by screening therapeutic compounds for early versus late passage organoids and, ultimately, a retrospective analysis of organoid response in vitro compared to primary tumor response would be more definitive.

We anticipate that standardized and easy-to-use tumoroid culture systems will promote their use in assays in which immortalized 2D cancer cell lines are commonly used. To this end, OncoPro medium is manufactured at GMP-compliant, ISO 13485-certified manufacturing sites, is functionally tested to ensure that each production lot enables tumoroid growth, and was designed to enable consistent tumoroid media preparation and compatibility with multiple cancer indications and formats during 3D cell culture. The serum- and conditioned-medium free formulation streamlined medium preparation and allowed for standardization between users and institutions and from lot-to-lot (Supplementary Fig. S7), a clear need in the cancer organoid field^15^. Suspension culture is emerging as a more accessible alternative to embedded tumoroid culture, which requires greater amounts of expensive matrix material, precise pipetting, and, in many cases, careful control of reagent temperature. In previous studies, tumoroids exhibited reduced or minimal growth in suspension without ECM proteins, though the addition of 2–5% volume/volume ECM potentiated growth^28,54–56^. These studies have demonstrated suspension culture of primary colorectal, metastatic colorectal, and esophageal tumoroids, typically for 2–4 weeks, with some examples of longer durations (20–25 weeks)^48,55,56^. Another approach is to encapsulate tumoroid or organoid cells in small droplets of ECM that float in suspension medium, which has been demonstrated in NSCLC and non-malignant intestinal organoids^57,58^. In our study, the addition of 2% BME generated a reliable suspension culture system suitable for long-term culture across a variety of cancer indications when used in conjunction with OncoPro medium. The time required for laboratory personnel to subculture tumoroids cultured in suspension decreased by approximately 2-fold compared to the embedded approach in our testing, facilitating maintenance of tumoroid models for downstream assays. Importantly, we demonstrated few changes at the transcriptome level between tumoroids in embedded or suspension culture. Potential downregulation in hypoxia-related signaling, cytokine-cytokine receptor interactions, and TNFα signaling in suspension compared to embedded culture formats (Supplementary Fig. S12) should be considered during assay design.

As one example of how tumoroid assays could be implemented, we engineered a tumoroid reporter line to express GFP and used that line to assess NK cell cytotoxicity in a 3D culture format (Fig. 8). Testing the cytotoxic potential on 2D models of solid tumor cancers does not adequately represent the physiological conditions with which NK cells will contend in vivo, namely dense tumor tissue and nutrient gradients^59,60^, which are better represented with tumoroid morphologies. However, dense BME domes may inhibit immune cell infiltration^61^, an issue which the suspension culture outlined here circumvents. That said, murine-derived BME may itself cause reactivity of CD4 + T cells^62^, and the microenvironment in which tumoroid and immune cell co-culture assays are performed should be carefully considered. In our experiments, multiplexed reporters enabled analysis of NK-cell mediated cytotoxicity by two signals, GFP reduction and increased caspase-3/7 activity, that were quantifiable and distinct from tumoroid-only, medium-only, and NK cell-only controls in our experiments. These techniques could be readily adapted to research on other modalities of cancer therapeutics.

Finally, multiple reports have demonstrated that drug response in patient-derived tumoroid models, at least in some cases, correlates highly with matched donor response during clinical treatment^29,48–52,63–68^. Advancements in functional precision oncology - where measurable improvements in patient outcome are increasingly being cited - have been hampered by difficulties in standardized approaches for tumoroid culture^9,69–71^. Given the high degree of success of short-term culture in OncoPro medium and high fidelity of tumoroids to patient material demonstrated here, its use as a standardized cell culture medium in functional testing applications warrants further exploration. The high degree of site-to-site reproducibility observed in our experiments suggests that standardized cell culture approaches will improve the comparability of results from assays using patient-derived cancer models in the near term, helping to drive its adoption in both drug discovery and functional precision medicine applications.

## Materials and methods

### Tumor dissociation and initial cell seeding

De-identified cryopreserved dissociated tumor cells or de-identified fresh tumor resection samples were purchased from commercial vendors (Discovery Life Sciences, Huntsville, AL or Cureline Translational CRO, Brisbane, CA) following collection from patients of at least 18 years of age under informed consent. Collections were made with Institutional Review Board (IRB) and Ethics Committee approved protocols in compliance with applicable regulations, requirements, and guidelines as described and managed by the cell and tissue vendors. Tumor resection samples were shipped in Gibco Hibernate-A Medium (Thermo Fisher Scientific, Waltham, MA, USA, cat# A1247-501) supplemented with, minimally, 1X Gibco GlutaMAX Supplement (Thermo Fisher Scientific, cat# 35050-061), 1X Gibco penicillin-streptomycin (Thermo Fisher Scientific, cat# 15140-122), and 100 µg/ml Primocin (InvivoGen, San Diego, CA, USA, cat# ant-pm-05) at 4^o^C. For some tissues, transport medium was further supplemented with 1X B-27 Supplement (Thermo Fisher Scientific, cat# 17504-044) and 10 µM Y27632 (Selleck Chemicals, Houston, TX, USA, cat# S1049).

Upon receipt, samples were immediately washed twice with a tissue wash buffer. Tissue wash buffer was composed of Gibco Advanced DMEM/F-12 (Thermo Fisher Scientific, cat# 12634-010), 1X GlutaMAX, 10 mM HEPES (Thermo Fisher Scientific, cat# 15630-130), 1X B-27 Supplement, 2X penicillin-streptomycin, 100 µg/ml Primocin, and 10 µM Y27632. Lung tumor samples HuLu051421 and HuLu051921 were minced, transferred to 50 ml conical tubes, resuspended in tissue wash buffer containing 0.5 U/ml collagenase type IV (Thermo Fisher Scientific, cat# 17104-019), and enzymatically dissociated for 30 min at 37^o^C in a 5% CO_2_ incubator on a CO_2_ resistant shaker (Thermo Fisher Scientific, cat# 88881101) set at 120 rpm. Colorectal sample HuCo031721 and endometrial sample HuEn041521 were minced, transferred to 50 ml conical tubes, resuspended in tissue wash buffer containing 100 U/ml collagenase type IV (Thermo Fisher Scientific, cat# 17104-019), and enzymatically dissociated for 1 h at 37^o^C in a 5% CO_2_ incubator on a CO_2_ resistant shaker set at 120 rpm. All other colorectal, breast, and endometrial samples were minced, divided into six roughly equal portions, and transferred to six gentleMACS C Tubes (Miltenyi Biotec, Auburn, CA, USA, cat# 130-093-237). Each tube was treated with different combinations of enzyme cocktails, at 2.5 ml of enzyme solution per tube diluted in tissue wash buffer, to maximize the likelihood that some viable cells were released. Enzymatic dissociation cocktails consisted of mixtures of 0-200 U/ml collagenase type I (Worthington Biochemical, Lakewood, NJ, USA, cat# LS004214), 0-200 U/ml collagenase type II (Worthington Biochemical, cat# LS004202), 0-200 U/ml collagenase type III (Worthington Biochemical, cat# LS004206), 0-200 U/ml collagenase type IV (Worthington Biochemical, cat# LS004210), 0–10 U/ml dispase II (Thermo Fisher Scientific, cat# 17105-041), 0–20 U/ml elastase (Worthington Biochemical, cat# LS002279), 0-200 U/ml hyaluronidase (STEMCELL Technologies, Vancouver, BC, CAN, cat# 07462), and 0-500 U/ml DNase I (Thermo Fisher Scientific, cat# AM2239), combinations of which were generated using the Custom Design tool in JMP18 (JMP Statistical Discovery, Cary, NC, USA) software. For some samples, the Tumor Dissociation Kit, human (Miltenyi Biotec, cat# 130-095-929) was prepared as instructed by the manufacturer and used for one of the six conditions, with enzymes dissolved in tissue wash buffer. For select endometrial samples, dissociation of some tumor fractions was performed in the absence of Y27632 or in the presence of 1X CultureCEPT Supplement (Thermo Fisher Scientific, cat# A56799). These colorectal, breast, and endometrial samples were enzymatically dissociated for 1 h at 37^o^C on a gentleMACS Octo Dissociator with Heaters (Miltenyi Biotec, cat# 130-096-427) using program 37C_h_TDK_1. Dissociation conditions for derived tumoroid models where fresh tissue was processed in our lab are detailed in Supplementary Table S10. Cells released from each dissociation cocktail were later combined as described below prior to seeding cells for tumoroid culture.

Following dissociation, cells were pelleted, washed twice with HBSS with no calcium, no magnesium, and no phenol red (Thermo Fisher Scientific, cat# 14175-095) supplemented with 10 µM Y27632, and resuspended in culture medium. Culture medium consisted of Gibco OncoPro Tumoroid Culture Medium (Thermo Fisher Scientific, cat# A57012-01; medium prepared from OncoPro Basal Medium, B-27 Supplement, OncoPro BSA, and OncoPro Supplement following manufacturer instructions) containing 10 µM Y27632, 2X penicillin-streptomycin, and 100 µg/ml Primocin. All cells dissociated from colorectal tumor samples were resuspended in this medium, while the medium was supplemented for the samples from lung, breast, and endometrial tumors as detailed in Supplementary Table S1. Specifically, the medium for lung cancer included 10 ng/ml Gibco Heat Stable (HS) FGF-10 recombinant protein (Thermo Fisher Scientific, cat# PHG0372), while medium for breast and endometrial cancer samples included 10 ng/ml HS FGF-10 plus 10 nM beta-estradiol (Fisher Scientific 501848155). Cells were then counted using the Vi-CELL BLU Cell Viability Analyzer (Beckman Coulter, Indianapolis, IN, USA, cat# C19196) using the following parameters: minimum diameter = 8 μm; maximum diameter = 60 μm, images = 100; cell sharpness = 7.0; minimum circularity = 1.0; decluster degree = high; aspiration cycles = 3; viable spot brightness = 55.0%; viable spot area = 5.0%; mixing cycles = 3. Each sample was counted in triplicate, and average viable cell numbers were averaged to calculate the total number of viable cells per sample. Cells from all dissociation conditions were combined prior to plating. Cells were plated at a concentration of 2.5 × 10^5^ viable cells/ml in OncoPro medium supplemented for the tissue type being processed as indicated in Supplementary Table S1, containing 10 µM Y27632, 2X penicillin-streptomycin, 100 µg/ml Primocin, and 2% (v/v) Gibco Geltrex LDEV-Free Reduced Growth Factor Basement Membrane Matrix (Thermo Fisher Scientific, cat# A14132-02) in Nunc non-treated multidishes (Thermo Fisher Scientific). Tumoroids were either continuously cultured in the suspension format or were embedded in Geltrex domes as described below after the first 2–7 days of culture.

Additional dissociated cells were collected for subsequent RNA and DNA isolation and classified as tumor samples during analysis for comparison of initial tumors to tumoroid cultures. Samples for RNA analysis were collected by resuspending dissociated tumoroid pellets (from pooled dissociation conditions, if applicable) in Invitrogen TRIzol Reagent (Thermo Fisher Scientific, cat# 15596-026) and storing at -20^o^C until RNA isolation. Dissociated cells for DNA profiling were pelleted, washed twice with DPBS with calcium and magnesium (DPBS (+/+); Thermo Fisher Scientific, cat# 14040-133), and pelleted again, with the cell pellet stored at -80^o^C until the time of DNA isolation. When sufficient tissue was available, minced tissue (pre-dissociation) was also collected for subsequent RNA isolation by submersion in TRIzol reagent, mechanical grinding with a pestle, and storage at -20^o^C, and for subsequent DNA isolation by flash freezing in liquid nitrogen and storing at -80^o^C. In general, P0 samples were cultured in suspension for 2–7 days prior to collecting cells and embedding in Geltrex matrix for several passages (details on embedded culture below; see also Fig. 1).

### Tumoroid culture

Tumoroids were cultured in OncoPro medium as instructed by the supplier. Briefly, dissociated tumoroids, either at the time of thaw or following dissociation from tumor material, were resuspended in OncoPro medium containing 10 µM Y27632 and supplemented for the cancer indications cultured as described in Supplementary Table S1. Niche factors added for specific cancer indications during culture were 10 ng/ml HS FGF-10 recombinant protein for lung and head and neck cancers, 10 ng/ml HS FGF-10 plus 10 nM beta-estradiol for breast and endometrial cancers, and 10 ng/ml HS FGF-10 plus 10 nM gastrin I peptide (Fisher Scientific, cat# 30–061) for pancreas cancer. OncoPro media formulations also included 100 µg/ml Primocin and 2X penicillin-streptomycin. Tumoroids were cultured in either suspension or embedded formats. For embedded culture in BME domes, cells were mixed with Geltrex matrix and OncoPro Tumoroid Culture Medium such that 50 µl of the final mixture contained 50,000 viable cells with a final Geltrex matrix concentration of 10 mg/ml. Geltrex matrix domes (50 µl/dome) were spotted on tissue culture plates before inverting plates and incubating for 30 min at 37 °C and 5% CO_2_ to allow for Geltrex matrix polymerization. Plates were returned to the normal orientation and overlaid with OncoPro medium supplemented for the given cancer indication as described in Supplementary Table S1 and containing 10 µM Y27632. For suspension culture, dissociated cells were seeded at concentrations of 0.125-0.3 × 10^6^ cells/ml in OncoPro medium supplemented for the given cancer indication as described in Supplementary Table S1 and containing 10 µM Y27632 in non-tissue culture treated plates or flasks, after which 2% v/v Geltrex matrix was added and mixed. Medium was exchanged every 2–3 days for both culture formats following protocols in the OncoPro medium user guide.

Routine monitoring of tumoroid size was performed with an Invitrogen EVOS XL Core Imaging System (Thermo Fisher Scientific, cat# AMEX1000) or Invitrogen EVOS M7000 Imaging System (Thermo Fisher Scientific, cat# AMF7000). Tumoroids were subcultured when tumoroid diameter reached 100–300 μm, usually every 7–10 days (Supplementary Figure S2). Tumoroids were collected, and culture vessels were washed with cold Gibco DMEM/F-12, GlutaMAX supplement (Thermo Fisher Scientific, cat# 10565-018), which was added to the cell solution. Tumoroids were washed with cold DPBS without calcium or magnesium (DPBS (-/-); Thermo Fisher Scientific, cat# 14109-144), after which the cell pellet was resuspended in room temperature Gibco StemPro Accutase Cell Dissociation Reagent (Thermo Fisher Scientific, cat# A11105-01) supplemented with 10 µM Y27632. Tumoroids were incubated in Accutase reagent for 10–20 min at 37 °C and 5% CO_2_ with periodic agitation. Tumoroids were then triturated 20 times with a P1000 pipet to mechanically disrupt the 3D structures. Dissociated tumoroids were counted in triplicate using the Vi-CELL BLU Cell Viability Analyzer using the parameters described above and plated based on viable cell count for suspension (of 0.125-0.3 × 10^6^ cells/ml) or embedded (50,000 viable cells per 50 µl dome of 10 mg/ml Geltrex) culture. Dissociated cells were sampled for subsequent RNA and DNA isolation at various passages during culture. Additionally, cell banks were generated throughout culture by resuspending dissociated tumoroids at 2 × 10^6^ cells/ml in Gibco Recovery Cell Culture Freezing Medium (Thermo Fisher Scientific, cat# 12648-010), freezing overnight at -80 °C in a Mr. Frosty Freezing Container (Thermo Fisher Scientific, cat# 5100-001), and storing in a liquid nitrogen dewar. Cryopreserved cells were thawed for culture according to the OncoPro medium user guide.

### Culture of commercially available tumoroid models

Tumoroid models procured from the National Cancer Institute Patient-Derived Models Repository (NCI PDMR; full tumoroid line names and details provided in Supplementary Table S7) were cultured in medium outlined in PDMR standard operating procedures, with formulations calling for L-WRN conditioned medium instead using 500 pM Gibco Wnt Surrogate-Fc Fusion Recombinant Protein (Thermo Fisher Scientific, cat# PHG0402), 500 ng/ml recombinant RSPO1 (Thermo Fisher Scientific, cat# 120-38-500UG), and 100 ng/ml recombinant noggin (Thermo Fisher Scientific, cat# 120–10 C-250UG). The 3dGRO human ISO72 colorectal cancer tumoroid line was purchased from Millipore Sigma (Burlington, MA, USA, cat# SCC507) and cultured in vendor-specified homebrew media. Detailed recipes are provided in Supplementary Table S6. Embedded cultures were performed via the same methods as tumoroids derived in OncoPro medium, with 50,000 dissociated tumoroid cells embedded in 50 µL Geltrex matrix droplets upon culture initiation and after subculturing. Tumoroids were monitored until tumoroid diameter was approximately 100–300 μm, after which tumoroids were dissociated using Accutase reagent, counted, and reseeded as described above. Where possible, cell pellets for subsequent DNA isolation or cell resuspensions in TRIzol reagent were collected as described above at the passages detailed in Supplementary Table S7. NCI PDMR tumoroids were also cultured in OncoPro medium in embedded or suspension formats. Tumoroid cells were either seeded in OncoPro medium upon thaw of the initially received cryopreserved cells; from internally produced, cryopreserved cell banks following an initial expansion in embedded culture using homebrew media; or, in select cases, from active culture in homebrew media (details in Supplementary Table S7). For mutational and gene expression analysis, cells from an initial cell bank (either directly as received from vendor or after establishment of an internal cell bank using vendor-recommended culture conditions; see Supplementary Table S6 and Supplementary Table S7 for details) were compared to tumoroids expanded from the initial bank in different arms of the study. Some cultures were not processed for RNA sequencing (details in Supplementary Table S7).

### 2D cell culture

Colorectal cancer cells Caco-2 (HTB-37), RKO (CRL-2577), SW48 (CCL-231), SW837 (CCL-235), SW948 (CCL-237), and SW116 (CCL-233) were purchased from ATCC (Manassas, VA, USA), thawed, and cultured in Gibco DMEM/F-12 (Thermo Fisher Scientific, cat #11320-033), supplemented with 10% Gibco fetal bovine serum (FBS), certified, One Shot format (Thermo Fisher Scientific, cat #A31604-01) and 1% penicillin/streptomycin (Thermo Fisher Scientific, cat# 15140-148). Cells were cultured in T75 flasks for 72 h and, after reaching 80–90% confluency, detached using 0.05% Gibco Trypsin-EDTA (Thermo Fisher Scientific, cat #25300-054). Cells were counted and preserved for RNA and DNA analysis as described above.

### Targeted genomic sequencing

#### DNA extraction, library preparation, and sequencing

Genomic DNA (gDNA) was isolated from the frozen cell pellets using the Invitrogen PureLink Genomic DNA Mini Kit (Thermo Fisher Scientific, cat# K182002) following the manufacturer’s protocol. Isolated DNA was quantified using the Invitrogen Qubit dsDNA High Sensitivity Assay Kit (Thermo Fisher Scientific, cat# Q32854). Sequencing libraries were prepared from 10 ng gDNA using the Ion Torrent Oncomine Comprehensive Assay v3C (OCAv3C) kit (Thermo Fisher Scientific, cat# A35806) and the Ion Torrent Ion Chef instrument model 4247 (Thermo Fisher Scientific, cat# 4484177) following manufacturer’s instructions (MAN0015885 (Revision C.0)) using 15 amplification cycles of 8 min each. The instrument library preparation protocol pools the 8 barcoded sample libraries together on an equimolar basis with expected total concentration around 100 pM. The concentration of the pooled library was measured using a High Sensitivity DNA ScreenTape (Agilent Technologies, Santa Clara, CA, cat# 5067–5584) and a 4200 TapeStation System bioanalyzer (Agilent Technologies). The pooled library was diluted to 50 pM in Invitrogen non-DEPC Treated, Nuclease-Free Water (Thermo Fisher Scientific, cat# AM9938). Libraries were templated and loaded onto Ion Torrent Ion 540 Chips (Thermo Fisher Scientific, cat# A27766) using the Ion Chef instrument and the Ion Torrent Ion 540 Kit-Chef (Thermo Fisher Scientific, cat# A30011) according to the manufacturer instructions (MAN0013432, Revision (E.0), MAN0010851 (Revision F.0)). Sequencing was performed using the Ion Torrent Ion GeneStudio S5 System model 7727 (Thermo Fisher Scientific, cat# A38194) according to the manufacturer instructions.

#### QC, filtering, and analysis

Ion Reporter Software v5.18 (Thermo Fisher Scientific) was used to map the data to the human genome assembly version 19 and perform downstream analysis. The Oncomine Comprehensive v3-w4.2-DNA-Single Sample analysis workflow was used to align raw reads to the reference genome and identify genomic variants. Coverage analysis reports from the Ion Reporter Software providing measurements of mapped reads, mean depth, uniformity, and alignment over a target region were used as quality assessment of the sequencing reactions.

#### SNV analysis

SNV calls with coverage ≥ 100 were used for correlation analysis. In cases where an SNV was identified in only 1 sample of a comparison, a value of 0 was assigned to that locus in the other sample to represent the reference call (0 frequency for no mutation present/identified at that locus); though in some cases this absence may be due to lack of sequencing depth, we chose the more stringent interpretation by using the 0 value. In cases where multiple samples are cross-correlated at once, the addition of 0 values can increase correlation values between similar samples that both lack mutations at loci present in unrelated samples; this can lead to higher correlation values between samples, as more donors are compared, for example, Fig. 2a colorectal donors D1-D3 versus Fig. 4C but not lung donors Fig. 2a D1 and D2 versus Fig. 4C. The allelic frequency of SNVs at each genomic loci for samples were correlated using Pearson’s correlation in JMP18. Pairwise correlation plots include density ellipses drawn to encompass approximately 90% of the data values and regression lines with confidence intervals from multivariate analysis. For base substitution analysis, the six major single base substitutions: C > A, C > G, C > T, T > A, T > C, T > G were counted using Microsoft Excel. Oncogenic driver mutations were identified using the inbuilt Oncomine Variants plugin 5.18 filter in the Ion Reporter software. For Supplementary Fig. S1, mutations were called using the Oncomine Extended filter 5.18 and counted.

#### CNV analysis

Unfiltered results from the Oncomine Comprehensive v3-w4.2-DNA-Single Sample analysis workflow were downloaded and sorted for CNV coverage. Estimated ploidy values were cross-correlated by genomic locus in JMP18.

### Tumor mutation load assay

Genomic DNA was isolated and quantified as described above. Sequencing libraries were prepared from 20 ng of gDNA using the Ion Torrent Oncomine Tumor Mutation Load Assay, Chef-ready kit (Thermo Fisher Scientific, cat# A37910) and the Ion Chef following manufacturer’s instructions (MAN0017042 (Revision C.0)) using 13 amplification cycles of 16 min each. Library quantification, templating, and sequencing were performed as described above.

### qPCR genotyping assays

Genomic DNA was isolated and quantified as described above. Purified DNA was amplified using Applied Biosystems TaqMan Genotyping Master Mix (Thermo Fisher Scientific, cat# 4371357) in the presence of Applied Biosystems TaqMan SNP Genotyping Assay, human (Thermo Fisher Scientific, cat# 4351379) using an Applied Biosystems QuantStudio 12 K Flex Real-Time PCR System (Thermo Fisher Scientific, cat# 4471087) according to manufacturer’s protocol. Both commercially available and custom designed primer and probe combinations were screened to identify mutations in the *APC* gene (Supplementary Table S5).

### Bulk RNA sequencing

#### RNA extraction, library preparation, and sequencing

Cell pellets were dissolved in TRIzol reagent and RNA was isolated using the Invitrogen PureLink RNA Mini Kit (Thermo Fisher Scientific, cat# 12183018 A). RNA integrity and concentration were determined using a High Sensitivity RNA Screen Tape (Agilent, cat# 5067–5579) and a 4200 TapeStation System bioanalyzer (Agilent); RNA concentration was quantified using the Invitrogen Qubit RNA High Sensitivity Assay Kit (Thermo Fisher Scientific, cat# Q32852). DNA digestion was performed using the Invitrogen ezDNase enzyme (Thermo Fisher Scientific, cat# 11766051). Complementary DNA (cDNA) was then synthesized from 10 ng RNA using the Invitrogen SuperScript IV VILO Master Mix kit (Thermo Fisher Scientific, cat# 11766050) or Invitrogen SuperScript VILO cDNA Synthesis Kit (Thermo Fisher Scientific, cat# 11754050) following the manufacturer protocol. Sequencing libraries were prepared from the total cDNA synthesis product using the Ion Torrent Ion AmpliSeq Transcriptome Human Gene Expression Panel, Chef-Ready Kit (Thermo Fisher Scientific, cat# A31446) and the Ion Chef instrument model 4247 following manufacturer’s instructions (MAN0010742 (Revision C.0)) and using 12–13, 16-minute amplification cycles. The pooled library concentration was measured using a High Sensitivity D1000 ScreenTape (Agilent, cat# 5067–5584) and a 4200 TapeStation System bioanalyzer (Agilent). The pooled library was diluted to 70 pM using Invitrogen non-DEPC treated, nuclease free water (Thermo Fisher Scientific, cat# AM9939). Templating and sequencing were performed as described above. In occasional cases of low read quality, reads could be increased by heat-denaturing an aliquot of the prepared sequencing library at 99^o^C for 5 min and then placing on ice for 2 min immediately before templating (updated guidance from MAN0013432 (Revision K.0)).

#### QC, filtering and analysis

 The sequencing data were automatically transferred to the S5 Torrent server virtual machine for alignment, quality control, and analysis using the human genome assembly 19 as the reference genome. Sequence read alignment was performed using the hg19 human reference file: ‘hg19_AmpliSeq_transcriptome_ercc_v1’. The AmpliSeqRNA plugin was used to target regions of the 21 K reference sequence genes using the hg19_AmpliSeq_Transcriptome_21K_v1 target panel to generate reads matrix data. Only samples with total reads above 5 million were considered for further downstream analysis. Differential gene expression analysis was performed using DESeq2 (version 3.19)^72^ using false discovery rate (FDR) *<* 0.05 and log2 fold-change (absolute value *>* 1) parameters to define differentially expressed genes (DEGs). DEGs were used for further downstream pathway enrichment analysis, performed using ShinyGO 0.77^73^. Enriched Kyoto Encyclopedia of Genes and Genomes (KEGG) pathways are displayed. In cases where more than 10 pathways were enriched, the top 10 results (ranked by FDR) are shown. FDR > 0.05 was used as a threshold to identify significantly enriched KEGG pathways. PCA analysis and sample-to-sample distance analysis were performed using variance stabilizing transformed (VST) data in DESeq2. For sample-to-sample distance analysis, the R function *dist* was used to calculate Euclidean distance between samples. For combined analysis of samples from multiple sequencing runs, gene expression data from all the cancer indications and donors were first normalized for sequencing depth, log_2_ transformed, and mean centered by genes (rows). Genes that had greater than 0.5 reads per million in at least two samples were filtered in to remove lowly expressed genes. The top 50 variable genes from the normalized gene expression data were calculated using the R function *var* and genes were clustered using hierarchical clustering.

### Molecular subtyping of initial cancer cells samples and tumoroids

#### Molecular subtyping of colorectal tumoroids

CRCAssigner, an R software package (https://github.com/syspremed/CRCAssigner), was used to predict consensus colorectal cancer molecular subtypes^19^. The package calculates the Pearson’s correlation between each sample expression profile and 38 marker genes (CRC-38) using the Prediction Analysis of Microarray (PAM) centroid method. Samples are subtyped into five major clinical subtypes, namely stem-like, inflammatory, transit amplifying, goblet-like, and enterocyte, based on the highest correlation. The PAM786 gene list^19^ was used for comparison of colorectal tumoroid lines to colorectal cancer tumor samples and established 2D colorectal cancer cell lines.

#### Molecular subtyping of breast tumoroids

PAM50, an R software package (https://github.com/ccchang0111/PAM50), was used to classify breast cancer samples into 5 major subtypes: Normal, Luminal B, Luminal A, Her2, and Basal-like based on gene expression levels of the PAM50 genes.

### Single-cell RNA sequencing

#### Library preparation and sequencing

Cryopreserved single cells from dissociated colorectal tumors and tumoroids were submitted to Azenta Life Sciences (Burlington, MA, USA) for scRNA-seq. The sample libraries were prepared in a single batch using the Chromium Single-Cell 3′ v3 Library and Gel Bead Kit (10x Genomics). Briefly, gel bead-based emulsions (GEM) were generated by combining barcoded single-cell 3′ Gel Beads, cells, and partitioning oil. Ten times barcoded, full-length cDNAs generated from GEMs were amplified by polymerase chain reaction (PCR). Enriched libraries were enzymatically digested, size selected, and adaptor ligated for sequencing. Sequencing libraries were generated with unique sample indices for each sample and quantified using the Kapa library kit. Quantified libraries were sequenced on an Illumina NovaSeq 6000.

#### QC, filtering and analysis

cellranger-7.1.0 Single-Cell Software Suite from 10x Genomics (https://www.10xgenomics.com/support/software/cell-ranger/latest) was used to align FASTQ sequencing reads to the cellRanger_GRCh38_v5 reference transcriptome, generating single-cell feature counts and associated unique molecular identifiers (UMIs) for each sample. The Seurat R package (version 4.3.0) was used to pre-process single-cell data. The CreateSeuratObject function was used to create Seurat objects for the count data. All the 10x genomics Seurat objects were merged using the merge function in Seurat 4.3.0. Genes/features shared by three or more cells, cells that expressed ≥ 200 and ≤ 5,000 genes, and mitochondrial gene content < 20% were included for analysis. The gene expression measurements for each cell were normalized by the total expression, multiplied by a scale factor of 10,000, and then log transformed. The FindVariableFeatures function was used to identify the top 2,000 highly variable genes. To scale the expression of each gene, data was transformed linearly so that the mean was 0 and variance was 1. PCA was performed on the scaled 2000 highly variable genes. Data were visualized using Uniform Manifold Approximation and Projection (UMAP). Cell clusters were identified by a shared nearest-neighbor (SNN) modularity optimization-based clustering algorithm set at a resolution of 0.5. To identify differentially expressed genes for cluster demarcation, the FindAllMarkers module was used, and genes expressed in more than 25% of the cells in each cluster were selected. The top 20 differentially expressed genes markers from each cluster were used to annotate cell types.

Gene expression data of 18,042 single cells from two colorectal tumors and tumoroids were aggregated first, and, using unsupervised clustering, cells were then clustered together in two-dimensional space. Epithelial cell adhesion molecule (*EPCAM*) was used to identify epithelial cell clusters. Collagen 1 A1 (*COL1A1*) was used to identify fibroblast cell clusters, and the mesenchymal marker vimentin (*VIM*) was used to identify stromal and immune cell clusters. Protein tyrosine phosphatase receptor type C (*PTPRC*) or leucocyte common antigen (*CD45*) was used to distinguish immune cells from stromal cells (Supplementary Fig. S4).

Raw gene read counts with pre-annotated cell types (immune cells, epithelial cells, endothelial cells, fibroblast cells) were transcript per million (TPM) normalized and log2 transformed for dissociated tumor cells (P0), tumoroids at passage 10 (P10) and tumoroids at passage 27 (P27) for HuCo3209 and HuCo021320 colorectal donors. In each donor, the genes across all the valid cells across passages with mean(log2(TPM)) > = 1.5 were selected to construct donor gene expression matrix *M* (*donor*) = genes×cells across different passages (P0, P10, and P27). Genes with enough reads were retained as initial input to infer Copy Number Variation (CNV) and were ordered by genomic coordinates from chr1 to chr22. Immune cells from the P0 cells were used as the “referenced group”, to infer CNV from EPCAM positive cells in matrix *M* (donor) as the “observed group”. Using R package inferCNV (https://github.com/broadinstitute/inferCNV) with default parameters compatible to 10x genomics scRNA-seq library, the CNV profile for each cell was estimated.

### In vivo tumorigenicity experiments and histology

Tumoroids were expanded in suspension culture in OncoPro medium per manufacturer’s instructions before being dissociated, counted, and resuspended in DPBS(+/+) containing 33% Corning Matrigel basement membrane extract (BME) such that a 100 µL injection volume contained 1e6, 3e6, or 5e6 cells. Cells were injected at 1e6, 3e6, or 5e6 cells per NSG mouse subcutaneously, with three animals per condition, at the University of Maryland Greenebaum Comprehensive Cancer Center Translational Laboratory Shared Service (TLSS) facility, and methods are reported following ARRIVE guidelines (https://arriveguidelines.org) to the best of our ability. Sample size was determined based on past experience in obtaining a reproducible mean tumor size by the TLSS facility. HuCo1044 tumoroids and HuLu051921 tumoroids were derived from female donors and injected into female mice. As this experiment was designed to test the feasibility of xenograft growth using tumoroid models, no control group was used. All experiments were performed under Institutional Animal Care and Use Committee (IACUC) approval by the University of Maryland Baltimore Animal Care and Use Program in accordance with relevant guidelines and regulations. Tumor volume was monitored for up to 90 days post-injection, at which time mice were euthanized by CO_2_ inhalation. Mice were euthanized prior to this time point if tumors reached 10% of mouse body weight, or if no tumor was present at 60 days post-injection. Researchers were not blinded to the tumoroid line which had been injected during monitoring. For the HuLu051921 line, all three tumors were not measured at each time point. Tumor volume in one animal was measured at the 11, 32, and 35 day time points, and the day 40 tumor volume measurement for HuLu051921 was made on two instead of three animals. Tumor formation was most robust at 3e6 cells per mouse injected, and data from that condition was used for Supplementary Fig. S6. Following euthanasia, tumors were excised and placed in fixative. Tumoroids from matched lines were cultured in suspension using OncoPro medium, collected, washed in cold DPBS (-/-), and fixed for 1 h at room temperature in Invitrogen Image-iT Fixative Solution (Thermo Fisher Scientific, cat# FB002). Fixed tumor samples and tumoroids were washed twice with DPBS (-/-) and then dehydrated by sequential washes with 30% ethanol, 50% ethanol, and 70% ethanol solutions (all diluted in water). Samples were stored in 70% ethanol solution prior to being processed for histology. Samples were paraffin embedded, sectioned, affixed to slides, and stained by Histoserv, Inc. (Rockville, MD, USA). Slides were stained with H&E, Jone’s PAMS, Movat Pentachrome, and Dane’s Method stains. Images of stained slides were acquired on an Invitrogen EVOS M7000 Imaging System.

### Generation of stable GFP-expressing tumoroid line

A transfer plasmid carrying an EmGFP gene under the EF1a promoter and a blasticidin resistance gene was transfected into human embryonic kidney (HEK) 293-derived Gibco Viral Production Cells using the Gibco LV-MAX Transfection Kit (Thermo Fisher Scientific, cat# A35684) to produce the lentivirus (LV) required for transduction. Prior to the transduction, HuCo1044 tumoroids were dissociated into a single-cell suspension. The number of viable cells was determined using the Invitrogen Countess 3 FL Automated Cell Counter (Thermo Fisher Scientific, cat# AMQAX2000). Cells were seeded into a 6-well plate at a seeding density of 2e5 viable cells per ml of OncoPro medium with a final volume of 2 ml per well. Transduction was performed in supplemented OncoPro medium at a multiplicity of infection (MOI) of 1, 5, 10, 25, and 50 in order to determine the optimal level. An MOI of 50 yielded the highest percent of GFP-positive cells as measured using an Invitrogen Attune NxT Flow Cytometer (Thermo Fisher Scientific, cat# A24858), and this sample was used for the rest of this study. Twenty-four hours post-transduction, cells were reseeded into standard suspension culture conditions in selection medium containing 5 µg/ml blasticidin. Cells were passaged every 7–14 days depending on tumoroid size, with media changes performed every 2–3 days. Four weeks after selection began, the cells were recharacterized by flow cytometry for GFP expression and by next generation sequencing using the Ion Torrent Oncomine Comprehensive Assay v3C and Ion Torrent Ion AmpliSeq Transcriptome Human Gene Expression Panel as described above.

### Flow cytometry for phenotyping of dissociated tumor cells and established tumoroids

Tumor samples (following dissociation of resection samples) or tumoroid cultures were dissociated as described above and passed through a 100 μm strainer. Briefly, cells were resuspended to 1e6 cells/ml in DPBS(+/+), and 1e4 to 1e5 cells were plated per well of a U-bottom 96-well plate. Cells were incubated with 1:500 Invitrogen LIVE/DEAD Fixable Yellow Dead Cell Stain (Thermo Fisher Scientific, cat# L34959) for 30 min at 4^o^C and then washed with Invitrogen eBioscience Flow Cytometry Staining Buffer (Thermo Fisher Scientific, cat# 00-4222-57). Cells were incubated with Invitrogen Fc Receptor Binding Inhibitor Polycolonal Antibody, eBioscience (FcBlock; Thermo Fisher Scientific, cat# 14-9161-73) at 20% concentration in Flow Cytometry Staining Buffer at 4^o^C for 20 min. Primary antibodies against surface targets were added and incubated for 1 h at 4^o^C. Antibodies were directed against EpCAM (1:20 final dilution; Thermo Fisher Scientific, cat# 25-9326-42, PE-Cyanine7), CD31 (1:20 final dilution; Thermo Fisher Scientific, cat# 46-0319-42, PerCP-eFluor), CD45 (1:10 final dilution; Thermo Fisher Scientific, cat# 12-0459-42, PE), and/or CEACAM (1:20 final dilution; Thermo Fisher Scientific, cat# 62-0668-42, Super Bright 436). Samples were washed three times with Flow Cytometry Staining Buffer and then fixed and permeabilized using the Invitrogen eBioscience Foxp3/Transcription Factor Fixation/Permeabilization Concentrated and Diluent (Thermo Fisher Scientific, cat# 00-5521-00) working solution according to the manufacturer’s protocol. Samples were blocked in 10% Gibco goat serum, New Zealand origin (Thermo Fisher Scientific, cat# 16210-064) in 1X Invitrogen eBioscience Permeabilization Buffer (Thermo Fisher Scientific, cat# 00-8333) for 15 min at room temperature. Intracellular antibody (against vimentin; 1:50 final dilution; Thermo Fisher Scientific cat# MA5-28601, APC) was added and incubated for 1 h at room temperature. Samples were washed twice and then resuspended in 200 µL Flow Cytometry Staining Buffer and analyzed on the Attune Nxt Flow Cytometer using the Invitrogen CytKick Autosampler (Thermo Fisher Scientific, cat#, A42901). Bead-based compensation (Invitrogen UltraComp eBeads Plus Compensation Beads, Thermo Fisher Scientific, cat# 01-3333-42 and Invitrogen ArC Amine Reactive Compensation Bead Kit, Thermo Fisher Scientific, cat# A10346) was applied and fluorescence minus one controls were used to set gates for expression.

### Preparation of the effector cells for NK-tumoroid co-culture

The natural killer (NK) cell line, NK-92, was procured from ATCC (CRL-2407) and cultured in Gibco RPMI 1640 Medium, GlutaMAX^T^ Supplement (Thermo Fisher Scientific, cat# 61870-036) + 10% Gibco fetal bovine serum, qualified, heat inactivated (Thermo Fisher Scientific, cat# 16140-071) + 10% Gibco horse serum, heat inactivated, New Zealand origin (Thermo Fisher Scientific, cat# 26050-088) + 500 U/ml Gibco recombinant human (rh) IL-2 (Thermo Fisher Scientific, cat# PHC0021). For primary NK cells, human leukopaks were procured from the San Diego Blood Bank and processed using the Gibco CTS Rotea Counterflow Centrifugation System (Thermo Fisher Scientific, cat# A50757/A50760). Primary NK cells were enriched by negative selection using the Invitrogen Dynabeads Untouched Human NK Cells Kit (Thermo Fisher Scientific, cat# 11349D) and expanded in Gibco CTS NK-Xpander Medium (Thermo Fisher Scientific, A50190-01) supplemented with 5% hAB serum (Fisher Scientific, cat# BP2525-100) and 500 U/ml rhIL-2, all according to manufacturer instructions. Primary NK cells were characterized via flow cytometry prior to initiation of the co-culture experiment.

### NK-tumoroid co-culture

Dissociated HuCo1044-GFP reporter tumoroid cells were seeded into a black-walled microwell plate with 400 μm diameter microcavities (Gri3D, Sun Bioscience, Lausanne, CH). Cells were seeded at 3 × 10^5^ viable cells per ml of supplemented OncoPro medium, 50 µL per well, and allowed to settle for one hour at 37ºC, 5% CO_2_. After the one-hour incubation, supplemented OncoPro medium + 2.67% v/v Geltrex matrix was added to each well to reach a final Geltrex concentration of 2% (v/v) and 10 µM Y27632. The cells were incubated and monitored for tumoroid formation for 3 days using an Incucyte SX5 Live-Cell Analysis System (Sartorius, Goettingen, DE, cat# 4816). On day 4, the effector cells (NK-92 or primary NK) were prepared for addition to the tumoroid culture at a variety of effector to target (E: T) ratios (0:1, 0.625:1, 1.25:1, 2.5:1, 5:1, and 1:0), wherein the number of target cells was estimated based on the known doubling time of the tumoroid line. The NK cells were resuspended so that the appropriate number of cells for a given E: T was contained in 50 µL to be added to each well, and cells were resuspended in NK-Xpander medium plus 5% hAB serum and 500 U/ml rhIL-2 for addition. Minimizing disturbance of intact tumoroids, as much medium as possible was removed from each well of the microwell plate. Effector cells were then added in 50 µL on top of the tumoroids. The plate was incubated for 1 h at 37ºC, 5% CO_2_ to allow the NK cells to settle. After incubation, an additional 150 µL of a 1:1 mixture of NK-Xpander medium (containing 5% hAB serum): OncoPro complete medium containing 500 U/ml rhIL-2 and 10 µM Invitrogen CellEvent Caspase-3/7 Red Detection Reagent (Thermo Fisher Scientific, cat# C10430) was added to each well. No Geltrex or Y27632 was added at the time of NK cell addition on day 4. The cells were incubated and monitored for GFP and Caspase-3/7 Red reagent signal for 3 days post-initiation of co-culture on the Incucyte platform. Average fluorescence intensity values were calculated in the Incucyte software from 3 replicate wells per condition.

## Electronic supplementary material

Below is the link to the electronic supplementary material.

Supplementary Material 1

Supplementary Material 2

Supplementary Material 3

## Data Availability

The datasets generated during the current study are available from the corresponding authors on reasonable request. Data used to generate figures have been uploaded to figshare at DOI: 10.6084/m9.figshare.26970553. The datasets generated using Illumina sequencing during the current study are available in the Gene Expression Omnibus (GEO), accession GSE285457.
